# New species and records of terrestrial slugs from East Africa (Gastropoda, Urocyclidae, Veronicellidae, Agriolimacidae)

**DOI:** 10.3897/zookeys.723.21817

**Published:** 2017-12-18

**Authors:** Ben Rowson, Megan Paustian, Jackie Van Goethem

**Affiliations:** 1 National Museum of Wales, Cathays Park, Cardiff, UK CF10 3NP; 2 Dept. Biology, Howard University, Washington, DC, USA; 3 Royal Belgian Institute of Natural Sciences, Rue Vautier, B-1000 Brussels, Belgium

**Keywords:** endemism, forests, Helicarionoidea, introductions, land-snails, Mollusca, taxonomy

## Abstract

New and little-known terrestrial slugs are dealt with based on extensive collections made in East Africa (Kenya, Tanzania, and Uganda) 1993–2007. This account deals primarily with larger species from forests in the Eastern Arc Mountains of Tanzania. In Veronicellidae, *Pseudoveronicella* Germain, 1908 is extended to Tanzania by P. (Hoffmannia) zootoca tanzaniensis **subsp. n.** in the Udzungwa Mts. In Urocyclidae, *Dendrolimax parensis* **sp. n.** is described from the Pare Mts. and *Leptichnoides avisexcrementis* **sp. n.** is described from the Uluguru Mts. In Urocyclinae, *Tanzalimax tattersfieldi* **gen. & sp. n.** is described from the Usambara Mts., *Tanzalimax seddonae* **gen. & sp. n.** from the Uluguru Mts., and *Udzungwalimax suminis* **gen. & sp. n.** from the Udzungwa Mts. In addition, the ill-defined genus *Atrichotoxon* Simroth, 1910 is discussed and the little-known *Dendrolimax leprosus* Pollonera, 1906 is reported from Uganda. In Agriolimacidae, a species of *Deroceras* Rafinesque, 1820 is reported for the first time from southern Tanzania. The taxonomic attribution and significance of each discovery is discussed.

## Introduction

Terrestrial slugs are often encountered in tropical Africa, but are less frequently collected than snails and in many ways more demanding to study. The native slug fauna of tropical Africa is dominated by genera of the helicarionoid Urocyclidae Simroth, 1888 (Urocyclinae sensu Van Goethem 1977), an apparently purely African group. Also native are the apparently far less diverse systellommatophoran slugs of the Veronicellidae Gray, 1840 (= Vaginulidae von Martens, 1866). Slugs of the limacoid Agriolimacidae Wagner, 1935 are represented in tropical Africa by several Ethiopian endemics, but otherwise only by a few records of introduced European species. Since the revisions of African Urocyclinae by Van Goethem (1977), African Veronicellidae by Forcart (1953), and the monograph of the Agriolimacidae by Wiktor (2000), studies on African slugs have been few. They have included revisionary and faunistic work (Herbert 1997, Verdcourt 2006, Rowson 2007, Muratov 2010) and species descriptions (Rowson et al. 2010, Rowson and Van Goethem 2012), mainly concerning the eastern half of Africa. DNA data support the relationship between urocyclid slugs and shelled Urocyclidae and their placement in the Helicarionoidea Bourguignat, 1877 (Herbert & Mitchell 2009). This paper reports on further new taxa and noteworthy slug records from East Africa, based on collections made in East African forests between 1993–2007 by, among others, P. Tattersfield, M. B. Seddon, C. F. Ngereza, C. N. Lange and B. Rowson and held at the National Museum of Wales, Cardiff, Wales, UK (NMW).

Many East African Urocyclidae are extraordinarily variable in colour and markings, and dissection is generally required to identify the tribe or genus. Spermatophores, when present, often help identify and (presumably) delimit species. Variation in internal morphology and spermatophores across the NMW collection often hints at East African species or subspecies complexes in some of the genera. This interpretation falls between that of Verdcourt (1960a, b, 1962, 1965) and Verdcourt and Polhill (1961) who described many species and subspecies, and Van Goethem (1977) who synonymised many of them under a few names. Both workers examined material from the volcanic Kenyan and Tanzanian highlands, and the forests of Uganda, from which earlier collecting had been more extensive (e.g. by Pollonera 1906, 1909, 1911). They saw relatively little from the forests of the Eastern Arc Mountains of Tanzania and Kenya, except for that collected by Verdcourt and co-workers in the 1950s and 1960s. Species endemism in the Eastern Arc is especially likely to be high, as it is amongst shelled molluscs (e.g. Rowson and Tattersfield 2013). Species complexes require much more detailed revision for which this material may be useful in future.

Despite this complexity it is also clear that additional species, some very distinct, remain undescribed especially in Tanzania. The generic placement of these is often difficult, with new Tanzanian species recently attributed to the tribes Dendrolimacini Van Goethem, 1977 and Upembellini Van Goethem, 1977 only with some circumspection (Rowson et al. 2010, Rowson and Van Goethem 2012). Such enigmatic taxa indicate that some of the deeper evolutionary relationships among East African slugs are yet to be resolved.

## Materials and methods

All animals were drowned in water and are preserved in 80% ethanol, sometimes methylated and/or with 2% propylene glycol; dimensions given are for material in preservation. The slug collection was reviewed and around 100 animals dissected in 2013 and 2014. Only material that could not be attributed to known, widespread species (following Van Goethem 1977) is listed here, unless the records extend ranges substantially. Grid references are in decimal degrees.

Paratypes have been deposited at the National Museums of Tanzania, Dar es Salaam, Tanzania (NMT) and Royal Belgian Institute of Natural Sciences, Brussels, Belgium (RBINS). Suprageneric classification agrees with Van Goethem (1977) and Bouchet and Rocroi (2005). Van Goethem (1977) is indispensible for redescriptions, details of previous citations and type material of Urocyclidae; note that most of Simroth’s types could not be found by Van Goethem, and have still not been found (e.g. in the collections at Berlin; Glaubrecht 2010, Glaubrecht and Zorn 2012).

### Abbreviations

ad. = adult slug; ag = albumen gland; am = ampulla of spermatophore; AK = A. Kisondella; AR = A. Robert; at = atrium; bc = bursa copulatrix; bd = bursa copulatrix duct; BHW = B. H. Warren; BR = B. Rowson; CFN = C. F. Ngereza; CNL = C. N. Lange; dg = digitiform glands; ec = epiphallic caecum; ep1 = epiphallus 1 (i.e. part between the calc sac or flagellum and the epiphallic caecum; ep1 is usually wound around the penis); ep2 = epiphallus 2 (i.e. part between epiphallic caecum and penis); cs = calc sac; FE = F. Ebonga; fl = flagellum; FR = Forest Reserve; juv. = juvenile or subadult slug; MBS = M. B. Seddon; mo = muscular organ between atrium and oviduct; NMT = National Museums of Tanzania, Dar es Salaam, Tanzania; NMW = National Museum of Wales, Cardiff, UK; NO = N. Otieno; NP = National Park; og = oviductal gland; ov = (free) oviduct; ot = ovotestis; pe = (free) penis; pr = penial retractor muscle; ps = penial sheath; PT = P. Tattersfield; RBINS = Royal Belgian Institute of Natural Sciences, Brussels, Belgium; vd = vas deferens; vg = vagina; vm = muscular part of vagina.

## Systematic part

### Clade Systellommatophora Pilsbry, 1948

#### Superfamily Veronicelloidea Gray, 1840

##### Family Veronicellidae Gray, 1840

###### Genus *Pseudoveronicella* Germain, 1908

####### Subgenus Hoffmannia Forcart, 1953

######## Pseudoveronicella (Hoffmannia) zootoca (Hoffmann, 1927)

######### Pseudoveronicella (Hoffmannia) zootocatanzaniensis subsp. n.

Taxon classificationAnimaliaSystellommatophoraVeronicellidae

http://zoobank.org/1AFD402A-BC98-4D86-B9A3-15971B789B21

Figs 1–2 , 21 , 27–29 , 45–49

########## Material.

TANZANIA: Holotype NMW.Z.2003.001.00030: 1 ad., Mt. Mwanihana FR (7.82°S, 36.83°E), Udzungwa Mts. NP, Kilombero District, forest at 1050 m alt., leg. BR, PT, MBS & CFN, 29 Jan. 2003 (sample 1050 misc). Paratype 1 NMW.Z.2003.001.00031: 1 ad., data as previous but 1200 m alt. (sample 1200P). Paratype 2 NMW.Z.2003.001.00032: 1 ad., data as previous but 1695 m alt. (sample 1695S).

Comparative material of P. (Pseudoveronicella) liberiana (Gould, 1850): UGANDA: 17 ads., Jubiya FR (0.27°S, 31.97°E), Masaka District, forest at 1180 m alt., leg. PT, BR, & FE, 3 Feb. 2007.

**Figures 1–10.**
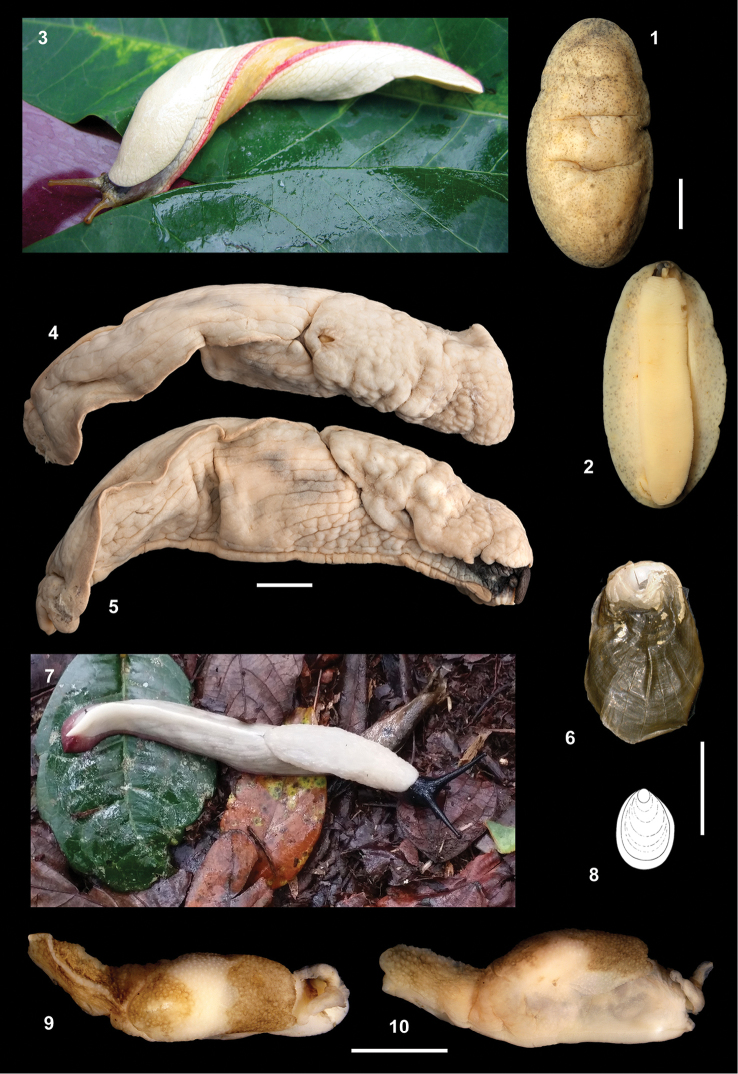
Living animals, habitus and shells. **1–2** Pseudoveronicella (Hoffmannia) zootoca tanzaniensis subsp. n., holotype **3** *Dendrolimax leprosus* Pollonera, 1906 at Jubiya **4–7** *Dendrolimax parensis* sp. n.: **4–5, 6** holotype **7** living animal at Kindoroko, not collected but probably this species **8–10** *Leptichnoides avisexcrementis* sp. n., holotype. Scale bars: 10 mm (**1–5**), 5 mm (**6–10**).

########## Description.

External appearance (Figs 1–2, 45). Medium-sized slugs (notum length 33–39 mm), notum weakly mottled grey-brown or grey-green, evenly speckled with small dark grey speckles, which continue onto the lighter-coloured hyponotum. Tentacles dark grey. Conspicuous anus in the form of a crescent-shaped slit with strongly flaring lips. Female genital opening just anterior to the mid-point of the right hyponotum. Juveniles not known.

Jaw and radula (Figs 21, 27–29). Similar to other African Veronicellidae: jaw of overlapping plates, accompanied by chitinous (?) bristles forming rows along the upper lip and two patches at the margins of the lower lips. Radula with central tooth and 47 teeth in a half-row. Central tooth small, rounded, stump-like; laterals simple, unicuspid and triangular; marginals subtriangular, becoming increasingly elongate and irregular in outline.

Genitalia (Figs 46–49). Penial sheath thin-walled, smooth, incorporating a verge and the conical tip of the “penial gland” of Forcart (1953). Verge small, lacking thorns or spines, basally swollen and with a single rim around the glandular, glans-like tip, which is symmetrical and lacks a fringe around its opening. Penial gland in the form of a smooth cone with an opening at its pointed tip. Five long, tangled digitiform glands enter the penial sheath near one another at the muscular base of the cone. Embryos not found; a single soft spheroidal mass (perhaps an egg) present in the uterus of one individual.

########## Etymology.

From Tanzania.

########## Distribution and habitat.

Apparently endemic to forest in the Udzungwa Mts. *Pseudoveronicella* is a West and Central African genus previously known in eastern Africa only from western Uganda and a single occurrence in Ethiopia (Forcart 1953, Verdcourt 2006, Wronski and Hausdorf 2010). It is easily recognised by the slit-like anus. Using Forcart (1953), all other Tanzanian Veronicellidae examined key to *Laevicaulis* Simroth, 1913, a genus widespread in eastern Africa (Forcart 1953, Herbert 1997, Verdcourt 2006), while the Ugandan material keys to *P. liberiana*. The Udzungwa specimens thus extend the range of the genus *Pseudoveronicella* far to the southeast, providing further evidence of western affinities among the endemic and other molluscs of these mountains (Rowson and Van Goethem 2012).

########## Remarks.

In Forcart (1953) the Udzungwa specimens key to Pseudoveronicella subgenus Hoffmannia. This differs from *Pseudoveronicella* *s. str.* (examined from Uganda) in having a verge that is glandiform rather than utricular, with an opening that is not surrounded by fringes. It then keys to P. (H.) zootoca, whose verge lacks thorns and has a glans-like tip. The shape of the tip differs between the two subspecies recognised by Forcart: P. (H.) z. zootoca, widespread in West Africa from Tamassadou to Leopoldville, and P. (H.) z. aethiopica Forcart, 1953 described from Sidamo, southern Ethiopia, at 2500 m. The Udzungwa slugs resemble P. (H.) z. aethiopica more closely than P. (H.) z. zootoca in their larger body size, and in not containing embryos (Forcart, 1953). However they do not confirm exactly to either: the verge has a much more strongly swollen base than either subspecies, while the glans is not hoof-like as in P. (H.) z. aethiopica. In light of the morphological differences and the greatly disjunct distributions, we follow Forcart (1953) in ascribing the material to a new subspecies of P. (H.) zootoca.

### Clade Stylommatophora Schmidt, 1855

#### Superfamily Helicarionoidea Bourguignat, 1877

##### Family Urocyclidae Simroth, 1888

###### Subfamily Urocyclinae Simroth, 1888

####### Tribe Dendrolimacini Van Goethem, 1977

######## Genus *Dendrolimax* Heynemann, 1868

######### Dendrolimax leprosus

Taxon classificationAnimaliaStylommatophoraUrocyclidae

Pollonera, 1906

Fig. 3

########## Material.

UGANDA: 9 ads., Jubiya FR (0.27°S, 31.97°E), Masaka District, forest at 1180 m alt., leg. PT, BR, & FE, 3 Feb. 2007.

########## Remarks.

This species keys unambiguously to *Dendrolimax* in Van Goethem (1977), who noted that all *Dendrolimax* other than *D. osborni* Pilsbry, 1919 were poorly-known. One such species is *D. leprosus*, previously known only from the type locality of “between Kijemula and Madudu”. According to Van Goethem (1977) this locality is at 0°41'N, 31°28'E (i.e. 0.68°N, 31.47°E). This is in Uganda approximately 100 km NE of Jubiya at a similar elevation (1300 m). Until now *D. leprosus* was known only from the types (which Van Goethem could not locate) and Pollonera’s (1906, 1909) description and figures. The Jubiya material corresponds well to these: individuals range from white to olive-coloured with large pale lesion-like blotches; the genitalia, jaw and shell are similar; and perhaps most distinctively, the radula has the majority of laterals bicuspid rather than tricuspid as in *D. osborni* (Van Goethem 1977).

The live animals were strikingly coloured in having a violet-pink foot-fringe. When handled, they secreted mucus of the same colour onto the hands, as if in defence. It appeared as though the mucus came from the foot-fringe itself rather than the supraperipodial grooves, which are conspicuous in *Dendrolimax* species (Van Goethem 1977).

######### Dendrolimax parensis sp. n.

Taxon classificationAnimaliaStylommatophoraUrocyclidae

http://zoobank.org/9CF1B989-D277-4219-8DCD-532EA483E7A9

Figs 4–7 , 22 , 30–32 , 50–54

########## Material.

TANZANIA: Holotype NMW.Z.1998.003.00002: 1 ad., Chome FR (4.30°S, 37.96°E), South Pare Mts., Same District, forest at 1875 m alt., leg. CFN & CNL, 15 Jan. 1998 (sample IC). Paratype 1 NMW.Z.1998.003.00003: 1 ad., data as previous. Paratype 2 NMW.Z.1998.003.00004: 1 ad., Kindoroko FR (3.75°S, 37.64°E), North Pare Mts., Mwanga District, forest at 1620 m alt., leg. MBS & CFN, 19 Jan. 1998 (sample IC). Paratype 3 NMT: 1 ad., data as previous but leg. PT & CNL (sample IIC). Excluded from type series: 3 ad. (dried out), data as previous but 1820 m alt.

########## Description.

External appearance (Figs 4–5). (In preservation; living appearance not recorded other than “reddish”, and “*Limax*-like”; but see Fig. 7). Very large (to 105 mm long), heavily-built slug, plain pale cream with black head and tentacles, lacking markings of any sort. Sole coloured as body, tripartite. Very strong, smooth, acute dorsal keel along whole length of tail, terminating in a short, blunt caudal appendage. Evident supraperipodial groove running parallel to strong peripodial groove as far as tail. Tail and flanks with large, smooth and fairly flat, tubercules. Mantle large (approx. 45% of body length) with cauliflower-like surface, with moderately-sized shell pore, attached at rear. Juveniles not known.

Shell (Fig. 6). Fingernail-shaped, symmetrical, to 9 mm long, thin and weakly mineralised around the nucleus only.

Jaw and radula (Figs 22, 30–32). Jaw with strong median projection. Radula with central tooth and up to 193 lateral and marginal teeth in a half-row, in about 150 rows. All teeth tricuspid but with mesocones pointed and by far the largest, other cusps tiny. No serrated outer edges to the outermost marginals.

Genitalia (Figs 50–51). Visceral cavity does not quite reach tail (posterior 15-20% of body solid). No stimulator, no calc sac. Atrium very short, with internal folds. Penial complex consisting of: stout free penis; moderately long flagellum (axial thread not found); short epiphallus 1 and epiphallus 2, approximately equal in length; moderately long epiphallic caecum. Penial retractor muscle arising from diaphragm. Penial papilla with a double wall, and a smaller papilla inside; free penis also with a penial sheath. Vagina present, rather-thick walled, with weak internal folds. Bursa copulatrix duct robust, long, not pigmented or ornamented, internally with weak longitudinal pilasters; bursa voluminous, thin-walled, rounded apically. Oviductal gland large, quite thick-walled. Ovotestis sited anterior to albumen gland, albumen gland extending to near tail.

Spermatophore (Figs 52–54). Three spermatophores from bursa of holotype, up to 30 mm long when coiled. Single short spur present near apical bend at junction between ampulla and tail. Ampulla smooth, slender, with 1.5–2 volutions, up to 25 mm long uncoiled. Tail thread-like, up to 35 mm long uncoiled, with a single keel of saw-like spines throughout.

########## Etymology.

From the Pare Mts.

########## Distribution and habitat.

Recorded from remnant forest above 1600 m in the North and South Pare Mts., to which it is likely to be endemic. Both Pare blocks are geologically part of the Eastern Arc chain, lying adjacent to the West Usambara Mts. (which are part of the chain) and Mt. Kilimanjaro (which is not). Verdcourt (2004) considered the Pares malacologically understudied despite their proximity to better-known areas, and there are no previous slug records from the area.

########## Remarks.

There are few Tanzanian species with which this large species can be confused. It keys to Upembellini or Dendrolimacini using Van Goethem’s (1977) key, based on the presence of a flagellum, the viscera almost reaching the tail, and the large size of the adult animal. The form of the jaw and radular teeth favour Dendrolimacini since there are more than Van Goethem’s maximum for Upembellini (120 teeth in a half-row). The vagina, large oviductal gland, and interior of the penis recall both *Dendrolimax* Heynemann, 1868 and the two species currently attributed to *Upembella*: *U. adami* Van Goethem, 1969 from south-eastern DR Congo and *U. nonae* Rowson & Van Goethem, 2012 from the Udzungwa Mts. The Pare species differs from both *Upembella* species in the much shorter flagellum, and from *U. nonae* in the simpler spermatophore. The most similar spermatophore figured by Van Goethem (1977) is that of the central African *Dendrolimax osborni* Pilsbry, 1919, although this apparently often lacks the apical spur on the spermatophore.

A photograph taken of a very large living slug in 2016 at Kindoroko FR (Fig. 7) may well show an example of *D. parensis*. Notably, the photograph indicates a violet mucus exuded from the tail (cf. *D. leprosus* above).

This slug may have a role in traditional medicine. MBS, PT & CFN (pers. comm.) were told while collecting that members of the Pare (Wapare) ethnic group sometimes apply the mucus from slugs to human skin as a treatment for burns. We do not know which species are preferred, but this very large species seems a likely candidate.

####### Tribe Upembellini Van Goethem, 1977

######## Genus *Leptichnoides* Van Goethem, 1975

######### Leptichnoides avisexcrementis sp. n.

Taxon classificationAnimaliaStylommatophoraUrocyclidae

http://zoobank.org/D8B7480E-B5ED-414B-8755-237474DDAF7A

Figs 8–10 , 23 , 33–35 , 55–58

########## Material.

TANZANIA: Holotype NMW.Z.1996.148.00032: 1 ad., Uluguru North FR (6.93°S, 37.7°E), Uluguru Mts., Morogoro District, forest above Tegetero village, approx. 1300 m alt., leg. PT, 22 Jan. 1996 (sample IC). Paratype 1 NMW.Z.1996.148.00033: 1 juv., data as previous but sample IIF. Paratype 2 NMW.Z.1996.148.00034: 1 juv., Kimboza FR (7.01°S, 37.78°E), Uluguru Mts., Morogoro District, lowland forest on dolomitic limestone, approx. 350 m alt., leg. PT, 19 Jan. 1996 (sample IB). Paratype 3 NMW.Z.1996.148.00040: 1 ad., data as previous but 20 Jan. 1996 sample IB. Paratype 4 RBINS.I.G. 33548/MT.3608: 1 ad., data as previous but sample IA.

########## Description.

External appearance (Figs 9–10). Small slug (to 23 mm long) with unusual colour pattern, imparting a resemblance to a bird dropping: pale cream with two thick grey-brown bands orientated across the dorsum (rather than parallel to it as is so common in slugs), leaving a pale saddle-like band across the mantle. Head and tentacles dark grey-brown, keel and sole pale. Strong dorsal keel along whole length of tail, terminating in a moderately long caudal appendage. Tail and flanks made granulose by raised tubercles. Mantle very large (approx. 50% of body length), with granulose surface, with large shell pore, attached at rear. Juveniles similarly coloured and proportioned.

Shell (Fig. 8). Fingernail-shaped, symmetrical, to 4.1 mm long, consisting of an extremely thin sheet of periostracum, weakly mineralised around the nucleus, adhering to the tissue below and easily torn during extraction.

Jaw and radula (Figs 23, 33–35). Jaw with weak median projection. Radula with central tooth and up to 55 lateral and marginal teeth in a half-row. All teeth strongly tricuspid, mesocones largest, ectocones larger than endocones. Weakly serrated outer edges to the outermost marginals.

Genitalia (Figs 55–56). Visceral cavity does not quite reach tail (posterior 10% of body solid). No stimulator, no calc sac. Atrium very short. Penial complex consisting of: stout free penis: moderately long flagellum; short epiphallus 1 and epiphallus 2, approximately equal in length; moderately long epiphallic caecum. The flagellum and caecum are loosely tangled together like a ball of wool until spread out during dissection. Penial retractor muscle short, arising from diaphragm. Free penis double-walled near the atrium, with a small conical papilla at the first bend of the penis. Vagina absent. Bursa copulatrix duct long, reaching albumen gland. Bursa spherical, thin-walled. A long, straight, muscular organ (characteristic of *Leptichnoides*) between atrium and oviduct, with a thick-walled sheath, entering atrium through a coarse papilla. Hermaphroditic duct extremely short, barely perceptible between spermoviduct and large ovotestis, which lies near rear of mantle. Albumen gland small.

Spermatophore (Figs 57–58). Single, partly-digested spermatophore from bursa. Apical bend at junction between ampulla and tail broken during manipulation. Ampulla smooth, thin-walled, 2.5 mm long, little coiled (less than 1 volution). Tail at least 1.6 mm long, of at least 1 volution, apically swollen then tapering. Strongly ornamented with a double keel of large, curved, apparently unforked spines.

########## Etymology.

From Latin *avis*, bird, and *excrementis*, faeces, in reference to the species’ resemblance to a bird dropping.

########## Distribution and habitat.

We initially suspected this species to be endemic to forest in the Uluguru Mts., where several such taxa occur (e.g. Verdcourt 2006, Tattersfield and Rowson 2011). However, apparently conspecific material has since been collected further south in Tanzania, in forest at approx. 80 m alt., Hippo Hole, 20 km west of Kirenjerange, Lindi Region (9.57°S, 39.28°E) (J. M. C. Hutchinson, pers. comm., 2017). The following additional material from the NMW collections is referred to *Leptichnoides verdcourti* (Forcart, 1967), suggesting that both species range from southern coastal Tanzania to the lower altitude forests of the Eastern Arc Mts.: TANZANIA: NMW.Z.1995.016.00013: 1 ad., Pindiro FR (9.53°S, 39.27°E), Kilwa District, coastal forest at 350 m alt., leg. PT, 26 Feb. 1995 (sample II). NMW.Z.1995.016.00014: 3 juvs., Ngarama FR (9.33°S, 39.33°E), Kilwa District, coastal forest at 400 m alt., leg. PT, 25 Feb. 1995 (sample II). NMW.Z.1997.007.00009: 1 juv., Sali FR (8.95°S, 36.40°E), Mahenge Mts., Ulanga District, montane forest at 960 m alt., leg. AK, NO, CFN, MBS & PT, 5 Feb. 1997 (sample II). NMW.Z.1997.007.00010: 1 ad.?, Mzelezi FR (8.79°S, 36.72°E), Mahenge Mts., Ulanga District, forest on dolomitic limestone at 645 m alt., leg. AK, NO, CFN, MBS & PT, 6 Feb. 1997 (sample IC). NMW.Z.2003.001.00033: 1 juv., Mkungwe FR (6.90°S, 37.91°E), Uluguru Mts., Morogoro District, submontane forest, approx. 900 m alt., leg. BR & CFN, 7 Feb. 2003 (sample I misc).

########## Remarks.

*Leptichnoides* has not previously been recorded from East Africa (Verdcourt 2006) and was until now known from a single species, *L. verdcourti* (Forcart, 1967) recorded from Mozambique, Zimbabwe, and (as an introduction) from Seychelles (Forcart 1967, Van Goethem 1977, Gerlach 2006). Van Goethem (1977) noted that an unnamed species from Comoros (“Species D”) might also belong to *Leptichnoides*. The long, straight muscular organ between the atrium and the oviduct is characteristic of the genus. In the studied collections, material from several eastern Tanzanian lowland localities appears referable to *L. verdcourti* so the genus is clearly well established in Tanzania. Here we describe *L. avisexcrementis* on account of its small size and markedly distinct colouration which allows it to be readily separated from Tanzanian and other material of*L. verdcourti*, in which the body is more conventionally patterned with dark brown irregular longitudinal bands and spots (as figured in Forcart 1967 and Gerlach 2006).

The new species is also the only *Leptichnoides* from which a spermatophore has yet been reported. Although partially digested, it differs remarkably from that of all other East African slugs in the form and large spines. Although the spines are unforked, there is a resemblance to the spermatophores of the less fully-limacised, West African slugs of the “*Estria-Rhopalogonium* group” of Van Goethem (1977) and of urocyclid semi-slugs (Van Mol 1970). The spermatophore and animal show some resemblance to that of the Comoros slug genus *Comorina* Simroth, 1910, but the sole species *C. johannae* Simroth, 1910 was said to have a substantial dart sac not present in any other flagellum-bearing members of the Urocyclinae; whether the “dart sac” is homologous with the muscular organ in *Leptichnoides* is uncertain. Van Goethem (1977) had no material of *Comorina*, and ranked it as *incertae sedis*, wondering whether it belonged in Urocyclidae or even whether the genitalia were correctly described by Simroth (1910). New material of *Comorina* is needed to resolve this.

####### Tribe Urocyclini Simroth, 1888

######## Genus *Atrichotoxon* Simroth, 1910

**Figures 11–20.**
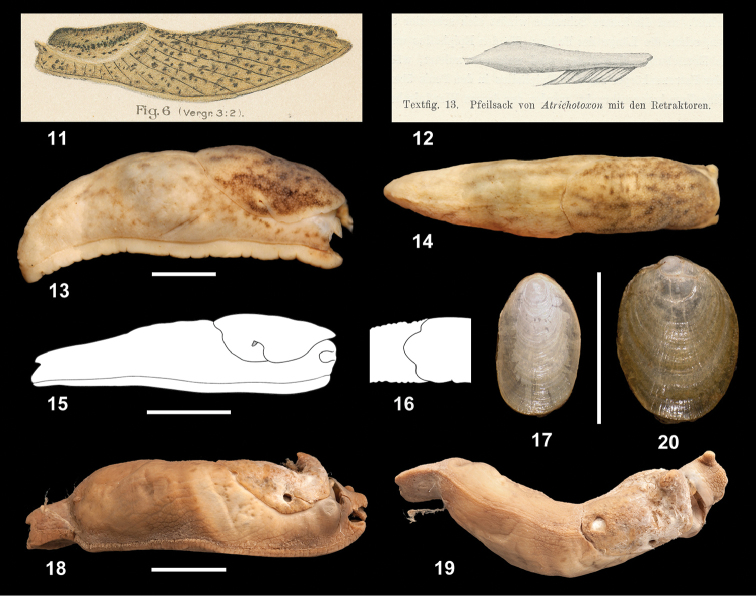
Habitus and shells, and dart sac. **11–12** *Atrichotoxon punctatum* Simroth, 1910: original figures of habitus and dart sac from Simroth (1910) **13–14 17** *Tanzalimax tattersfieldi* gen. & sp. n., holotype **15–16** *Tanzalimax seddonae*, gen. & sp. n., Paratype 1 **18–20** *Udzungwalimax suminis* gen. & sp. n., holotype. Scale bars: 10 mm (**13–16, 18–19**), 5 mm (**17, 20**).

The collections from different ranges of the Eastern Arc mountains include numerous large Urocyclini which lack darts. Using Van Goethem (1977), these features allow them to key to *Atrichotoxon* Simroth, 1910, although the species are evidently new. However, as explained below, *Atrichotoxon* is so problematic that we refer them to other, better-defined genera. It is important to quote from Simroth’s original text on *Atrichotoxon* because different interpretations of his descriptions have been made. The genus was introduced for a single species, *A. punctatum* Simroth, 1910, which he illustrated in colour (Simroth 1910: Taf. 26 Fig. 6; reproduced here as Fig. 11). It was said to be the ‘smallest *Trichotoxon*’ at 5 cm long, with a grey-brown background with dark mantle bands and grey to black spots.

Simroth’s internal description of *Atrichotoxon* (from *A. punctatum*) was clear about the similarities and differences from *Diplotoxon* Simroth, 1897, namely its type species Trichotoxon (D.) voeltzkowi Simroth, 1910. That species, from Pemba I., is now considered a synonym of the widespread *T. heynemanni* (Van Goethem 1977). The diagnostic features of *Atrichotoxon* all relate to the dart sac, the only part of the internal anatomy Simroth figured (1910: Textfig. 13; reproduced here as Fig. 12). This was said to be externally like that of *Diplotoxon*, apart from the strong bundles of retractors attaching it to the floor of the body cavity (“Ausserlich unterscheidet er sich durch einen kräftigen, aus vielen Bündeln zusammengesetzten Retraktor, der ihn am Boden der Leibeshöhle festheftet;”). The interior of the (primary) dart sac further resembles that of *Diplotoxon* in consisting of two (secondary) dart sacs of longitudinal muscle (“Wir sehen die beiden starken, aus Längsmuskeln aufgebauten sekundären Pfeilsäcke”). However, there were no darts, nor broken stumps indicating that darts had once been present. Simroth discounted that the darts had been ejected during mating (“Da das Tier völlig unverletzt war und da die Pfeile von *Trichotoxon*, wie ihre von der behaarten Scheide auch an der Bruchfläche überzogenen Stümpfe beweisen, höchstens abgebrochen, niemals aber, wie bei *Helix*, ausgestossen und erneuert werden, so scheint es auch hier ausgeschlossen, dass sie bei der Copula entfernt wären.”). Thus, *Atrichotoxon* was not simply a *Diplotoxon* which had lost or failed to develop its darts, but a species with a fully-formed, internally subdivided, yet empty dart sac with strong retractors.

Simroth’s figure did not, however, make it clear to which end of the strongly asymmetrical dart sac the muscles are attached. In the text, Simroth described the dart sac retractors as “pointing in another direction” (“Auch deutet der Retraktor in anderer Richtung.”). He explained this by suggesting that the entire dart sac was everted at mating into the body of the partner (“Hier hat offenbar das Rätsel, das ich oben beim *Tr. Voeltzkowi* berührte, wie der Penis zu dem oben in den Pfeilsack mündenden Bursagang gelangte, wie weit der Pfeilsack etwa ausgestülpt würde, seine radikale Lösung gefunden: Der ganze Pfeilsack wird ausgestülpt und wirkt nicht mehr als Reizorgan, sondern dient zur Vereinigung der Partner.”). The term “another direction” unfortunately still allows for interpretation, given the uncertain orientation of Simroth’s drawing. It is likely that he meant that the dart sac’s retractors would withdraw it into the body after mating, and so must contract away from the genital orifice (as appears to be the pattern in other Urocyclidae). However this would mean that the atrium is at the right-hand side of his drawing, and that the narrow end with retractors is the basal (atrial) end of a dart sac that is swollen distally. Either this situation or its alternative would make *Atrichotoxon* unique (in addition to the lack of darts). None of the taxa in Van Goethem’s (1977) monograph have either a distally swollen dart sac, or a proximally swollen one with retractors confined to its distal end.

Unfortunately, Simroth gave no more internal details and no locality other than East Africa (“Aus Ostafrika, von Stuhlmann erbeutet”). As with many of Simroth’s types, the types of *A. puncatum* are presumed lost (Van Goethem 1977, Glaubrecht 2010, Glaubrecht and Zorn 2012). Verdcourt’s three-part biography of F. L. Stuhlmann (Verdcourt 1988, 1989a, b) included a list of taxa collected by Stuhlmann that were possibly present in the Berlin collections in 1959. However, in the list all the slug taxa described by Simroth were marked with a †, presumably indicating either that they were not seen in 1959, or that they were later discovered to be lost. Although some of the Stuhlmann–Simroth veronicellid slugs from this list have since been rediscovered in Berlin, none of the urocyclids have (Glaubrecht 2010). Stuhlmann collected widely in East Africa, but spent several years (1903–1907) in the nearby East Usambaras as the director of the research station at Amani (Verdcourt 1988, 1989a, b). Verdcourt himself later spent a year (1949–1950) at the Amani station and described the slugs of the area, yet did not find *A. punctatum*. Although he also worked in the West Usambaras he spent much less time in this area. Having failed to find the species, Verdcourt later (2006) suggested the true origin of *A. punctatum* might be in either Tanzania or Uganda. However, five of the six other East African urocyclids in Simroth’s (1910) paper were from Tanzanian localities. The sixth, *Atoxon martensi* Simroth, 1910 was another single specimen collected by Stuhlmann from “East Africa” without further locality. Its description was even briefer than that of *A. punctatum*, and it was considered a species inquirendum by Van Goethem (1977). Verdcourt (2006) concluded that *A. martensi* was probably from Tanzania. Given also that all other records of the genus *Atrichotoxon* are from the Eastern Arc Mts. it thus seems more likely that *A. punctatum* was first collected in Tanzania than in Uganda.

Current usage is the second reason why *Atrichotoxon* is problematic. Verdcourt & Polhill (1961) and Verdcourt (1965) attributed material of a highly distinctive new species, *A. usambarense* (Verdcourt, 1961) to *Atrichotoxon*, which they then treated as a subgenus of *Trichotoxon* Simroth, 1888. A subsequent study (Van Goethem 1977) and the present material confirm the distinctness of *A. usambarense* and suggest it is restricted to the E. Usambara Mts. Verdcourt and Polhill (1961) and Verdcourt (1965) also discussed an *Atrichotoxon* “sp. ?n.” from Vuria Peak in the Bura/Taita Hills, Kenya, also part of the Eastern Arc. The dart sac of this species differed from *A. usambarense* in containing “many small splinter-like crystals in the convolutions which seem to be arranged radially and not facing down the cavity like darts.” They had no new material or localities for *A. punctatum*, but from Simroth’s publication described the dart sac as being “narrow, swollen distally and with retractors at the proximal cylindrical end” (p. 30). By “proximal” they evidently meant “basal”, i.e. near the atrium (p. 31). However, as they made clear (p. 30–31) neither of their *Atrichotoxon* species showed these retractors, and their figures show the dart sacs swollen basally, not distally. Despite this Verdcourt (1965) suggested the presence of atrial retractor muscles might diagnose *Atrichotoxon*. Van Goethem (1977) showed that such retractors also occur in some species of *Atoxon* Simroth, 1888 including the widespread *Atoxon pallens* Simroth, 1895, with which he synonymised another “*Atrichotoxon*” species, T. (A.) impressum Verdcourt, 1965 from Nairobi.

Although imperfect, this usage was followed by Van Goethem (1977) and subsequent works (e.g. Verdcourt 2006). Numerous specimens in the present collections, in several genera, have an external resemblance to *A. punctatum*, but we found none that match its characteristic internal anatomy (moreover, all *Trichotoxon* specimens investigated contain darts). We therefore propose to maintain the usage of Verdcourt (2006) of *Atrichotoxon* for *A. puncatum* and *A. usambarense* until authentic material of *A. punctatum* is found and properly studied. The following three species are therefore referred to other genera whose genital anatomy is fully described.

######## 

######### Genus Tanzalimax gen. n.

Taxon classificationAnimaliaStylommatophoraUrocyclidae

http://zoobank.org/B1F2D332-E12F-4E4F-9613-FBEA868441C5

########## Type species.

*Tanzalimax tattersfieldi* sp. n.

########## Included species.

*Tanzalimax tattersfieldi* sp. n. and *Tanzalimax seddonae* sp. n.

########## Diagnosis.

Slug belonging to tribe Urocyclini. Genital apparatus without an accessory organ (atrial diverticulum, sarcobelum or dart sac); penis muscular; penial tube thick-walled with narrow lumen; penial sheath muscular over its whole length; very distinct transition between epiphallus 2 and penis. Vagina with a strong muscular bulb; oviductal gland present. Spermatophore with at least one toothed keel at the tail tip. Radula: median and lateral teeth tricuspid, outermost laterals and marginals bicuspid or serrated.

**Figures 21–26.**
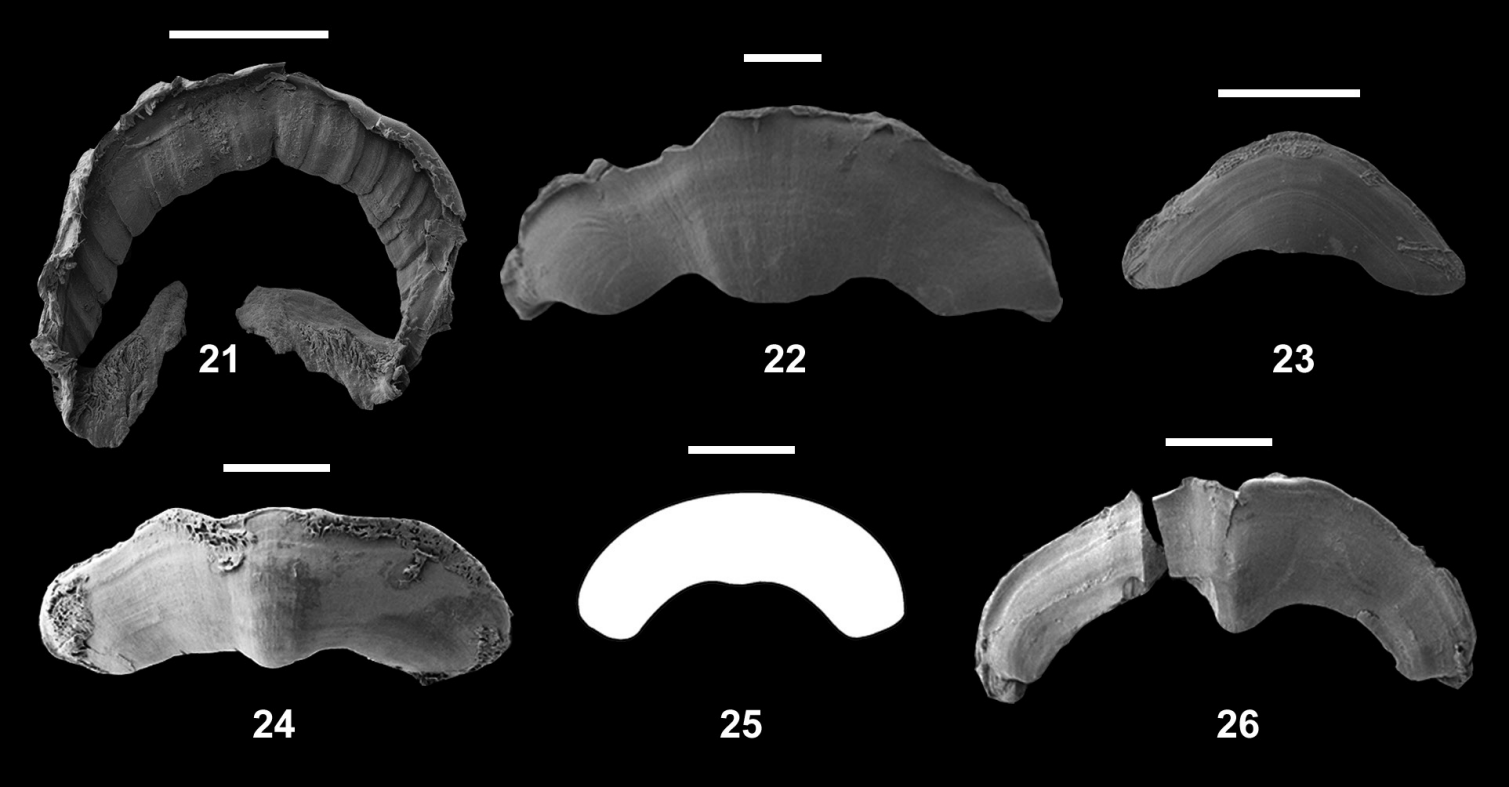
Jaws. **21** Pseudoveronicella (Hoffmannia) zootoca tanzaniensis subsp. n., Paratype 1., also showing patches of bristles **22** *Dendrolimax parensis* sp. n., holotype **23** *Leptichnoides avisexcrementis* sp. n., holotype **24** *Tanzalimax tattersfieldi* gen. & sp. n., holotype **25** *Tanzalimax seddonae*, gen. & sp. n., Paratype 1. **26** *Udzungwalimax suminis* gen. & sp. n., holotype. All scale bars: 1 mm.

########## Etymology.

From Tanzania and Latin *limax*, a slug (no relationship to *Limax* Linnaeus, 1758 is implied).

########## Known distribution.

Forest in the East and West Usambara Mts. and the Uluguru Mts. of the Eastern Arc Mts., Tanzania.

########## Gender.

Masculine.

########## Remarks.

See also remarks on *Atrichotoxon* and *Udzungwalimax* gen. n. below. The schematic of the structure of the genitalia of this genus (Figs 61–62) can be contrasted with those in Van Goethem (1977: 28). The monotypic *Phaneroporus* Simroth, 1888 from the coasts of Lake Tanganyika (south-western Tanzania and northern Zambia) is similar to *Tanzalimax* gen. n. and *Udzungwalimax* gen. n. in some respects, but differs in its massively enlarged penis and penial stylet (Van Goethem 1977).

######### Tanzalimax tattersfieldi sp. n.

Taxon classificationAnimaliaStylommatophoraUrocyclidae

http://zoobank.org/34B2EE14-347F-46E8-BB1C-CFDF75C9D47B

Figs 13–14 , 17 , 24 , 36–38 , 59–63 , 64–65

########## Material.

TANZANIA: Holotype NMW.Z.1996.148.00035: 1 ad., Ambangulu FR (5.08°S, 38.43°E), West Usambara Mts., Lushoto District, montane forest at approx. 1240 m alt., leg. PT, 29 Jan. 1996 (sample IIC). Paratype 1 RBINS.I.G. 33548/MT.3609, 1 ad., Bomole FR (5.1°S, 38.62°E), East Usambara Mts., Muheza District, forest at 1240 m alt., leg. PT, 4 Mar 1995 (sample II). Paratype 2 NMW: 1 ad., Mtai FR (38.46°E, 4.51°S), East Usambara Mts., Muheza District, leg. Frontier Tanzania, 1996.

**Figures 27–35.**
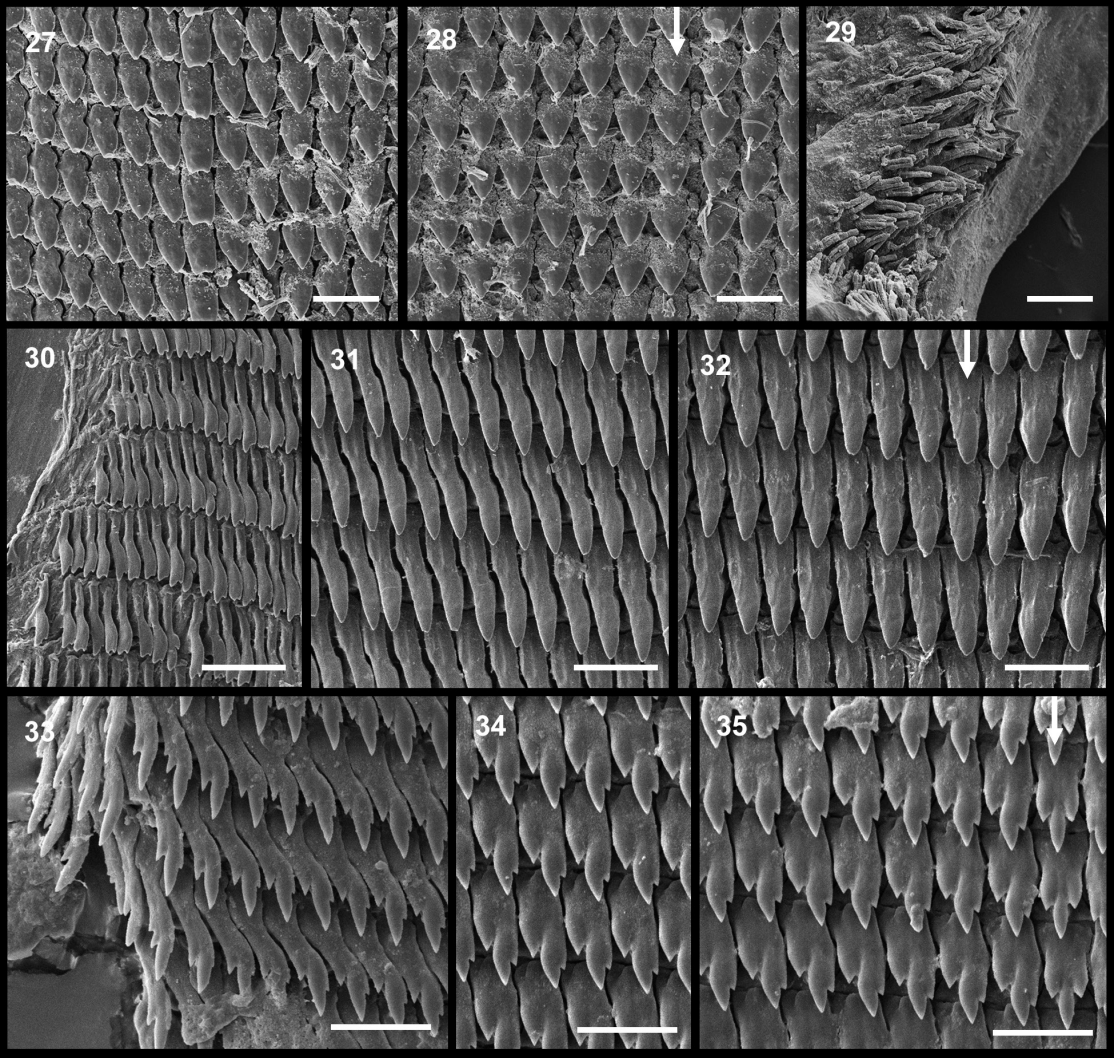
Radulae. From left to right, marginal, lateral and central teeth (arrow), each shown to the same scale. **27–28** Pseudoveronicella (Hoffmannia) zootoca tanzaniensis subsp. n., Paratype 1 **29** bristles from beneath jaw **30–32** *Dendrolimax parensis* sp. n., holotype **33–35** *Leptichnoides avisexcrementis* sp. n., holotype. All scale bars 50 μm except for **33–35**, 20 μm.

########## Description.

External appearance (Figs 13–14). (In preservation; living appearance not recorded). Medium-sized (30–40 mm long) slug, body, head and tentacles pale buff, well-marked with scattered dark brown spots on back and flanks, coalescing to form diffuse bands on mantle. Sole coloured as body, tripartite. Moderate dorsal keel along whole length of tail, terminating in a very short, blunt caudal appendage. Supraperipodial groove not evident. Tail and flanks with moderately large, but smooth and very flat tubercules. Mantle moderately sized (approx. 40 % of body length) with finely granular surface and large shell pore, attached at rear.

Shell (Fig. 17). Fingernail-shaped, symmetrical, 5.0 mm long, thin and weakly mineralised, with periostracum just extending beyond the margins.

**Figures 36–44.**
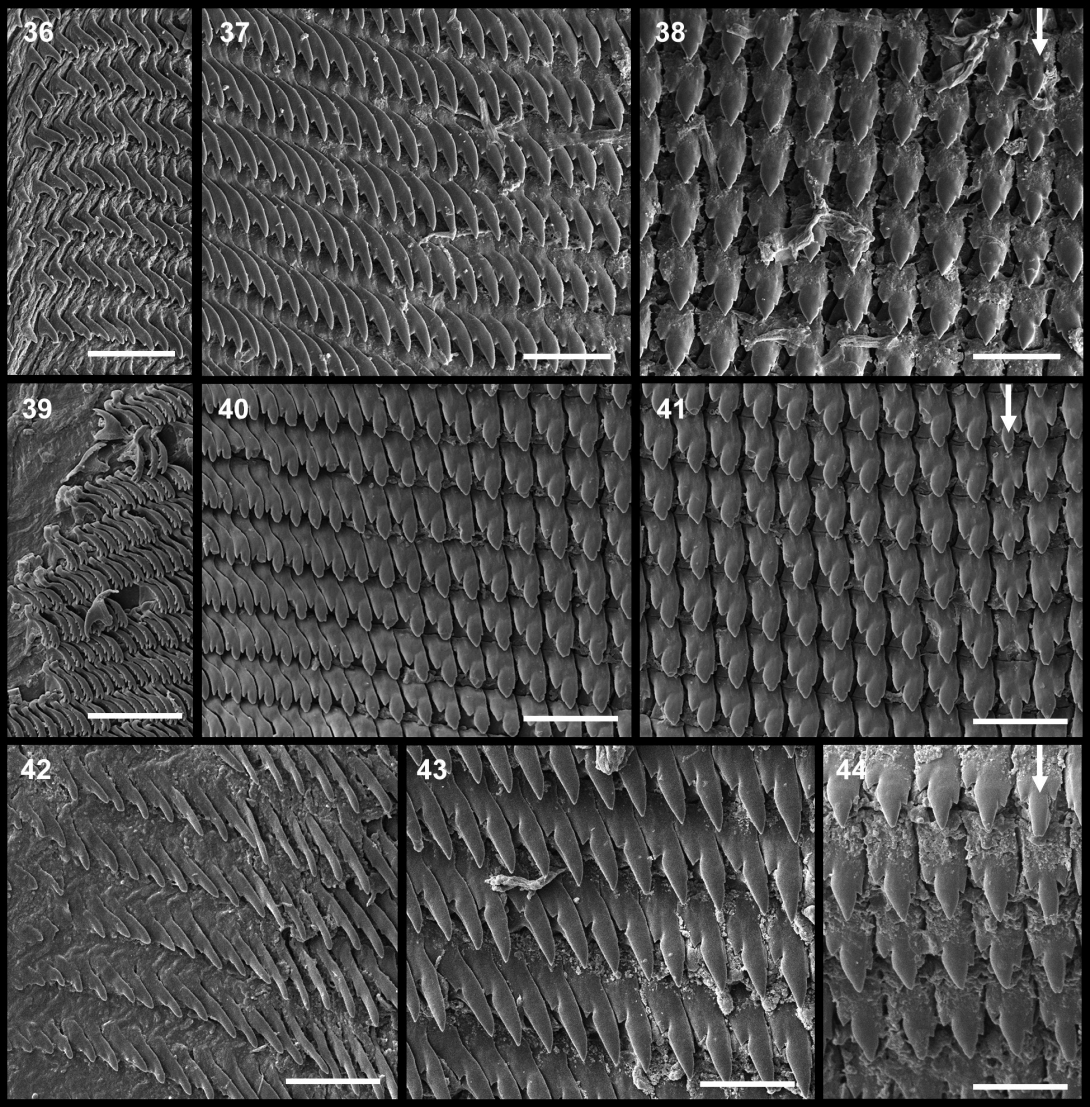
Radulae. From left to right, marginal, lateral and central teeth (arrow), each shown to the same scale. **36–38** *Tanzalimax tattersfieldi* gen. & sp. n., holotype **39–41** *Tanzalimax seddonae*, gen. & sp. n., Paratype 1 **42–44** *Udzungwalimax suminis* gen. & sp. n., holotype. All scale bars 50 μm.

Jaw and radula (Figs 24, 36–38). Jaw with strong median projection. Radula with central tooth and up to 80 lateral and marginal teeth in a half-row, in over 100 rows. Lateral teeth tricuspid, outermost laterals becoming bicuspid, but with mesocones pointed and largest. Serrated outer edges to some outermost marginals.

Genitalia (Figs 59–62, 64–65). Visceral cavity almost reaches tail (only the posterior 10% of body solid). No stimulator. Penial complex consisting of: strongly twisted free penis; epiphallus 1 long, epiphallus 2 very short; long epiphallic caecum; and pyriform calc sac. Penial retractor muscle arising from diaphragm. Internally, penis smooth. No penis verge. Moderately thick penial sheath present. Atrium long, with weak internal folds. Vagina with a strong muscular swelling at one side, internally with strong irregular folds and a sphincter near the swelling. Bursa copulatrix duct robust, long, not pigmented or ornamented, internally with weak longitudinal pilasters; bursa voluminous, thin-walled, pointed apically. Oviductal gland quite large, oviduct short and broad. Ovotestis sited posterior to albumen gland.

Spermatophores. Two fragmented spermatophore tails from bursa of holotype, toothed at the tail tip; three spermatophores from Paratype 2, up to 20 mm long.

########## Etymology.

Named in honour of Peter Tattersfield, in recognition of his work, encouragement and support of others in the study of East African terrestrial molluscs, including collecting many of the slugs studied here.

########## Distribution and habitat.

Forest in both the West and East Usambara Mts., where numerous endemic molluscs are known (e.g. Verdcourt 2006).

########## Remarks.

See *T. seddonae* sp. n.

######### Tanzalimax seddonae sp. n.

Taxon classificationAnimaliaStylommatophoraUrocyclidae

http://zoobank.org/28FCB609-3E33-42FA-B1A7-27693596AFD1

Figs 15–16 , 25 , 39–41 , 66–70

########## Material.

TANZANIA: Holotype RBINS.I.G. 33548/MT.3610, 1 ad., Uluguru North FR (6.93°S, 37.7°E), Uluguru Mts., Morogoro District, forest above Tegetero village, approx. 1300 m alt., leg. PT, 22 Jan. 1996 (sample IIG). Paratype 1 NMW.Z.1996.148.00041, 1 ad., data as previous but sample III.I. Paratype 2 NMW.Z.1996.148.00042, 1 juv., data as previous but sample ID.

########## Description.

External appearance (Figs 15–16). (In preservation). Medium-sized slug (holotype 39 mm, paratype 30 mm), body unicolourous ivory. Sole coloured as body, tripartite. Supraperipodial groove distinct. Tail long and rounded, with a blunt keel only at the end, terminating in a very distinct caudal appendage. Tail and flanks with a warty appearance. Mantle moderately sized (40% of body length) with fine granular surface; no shell pore.

Shell. Fingernail-shaped, symmetrical, 4.3 mm long, very thin, not mineralised, except for the apex.

**Figures 45–49.**
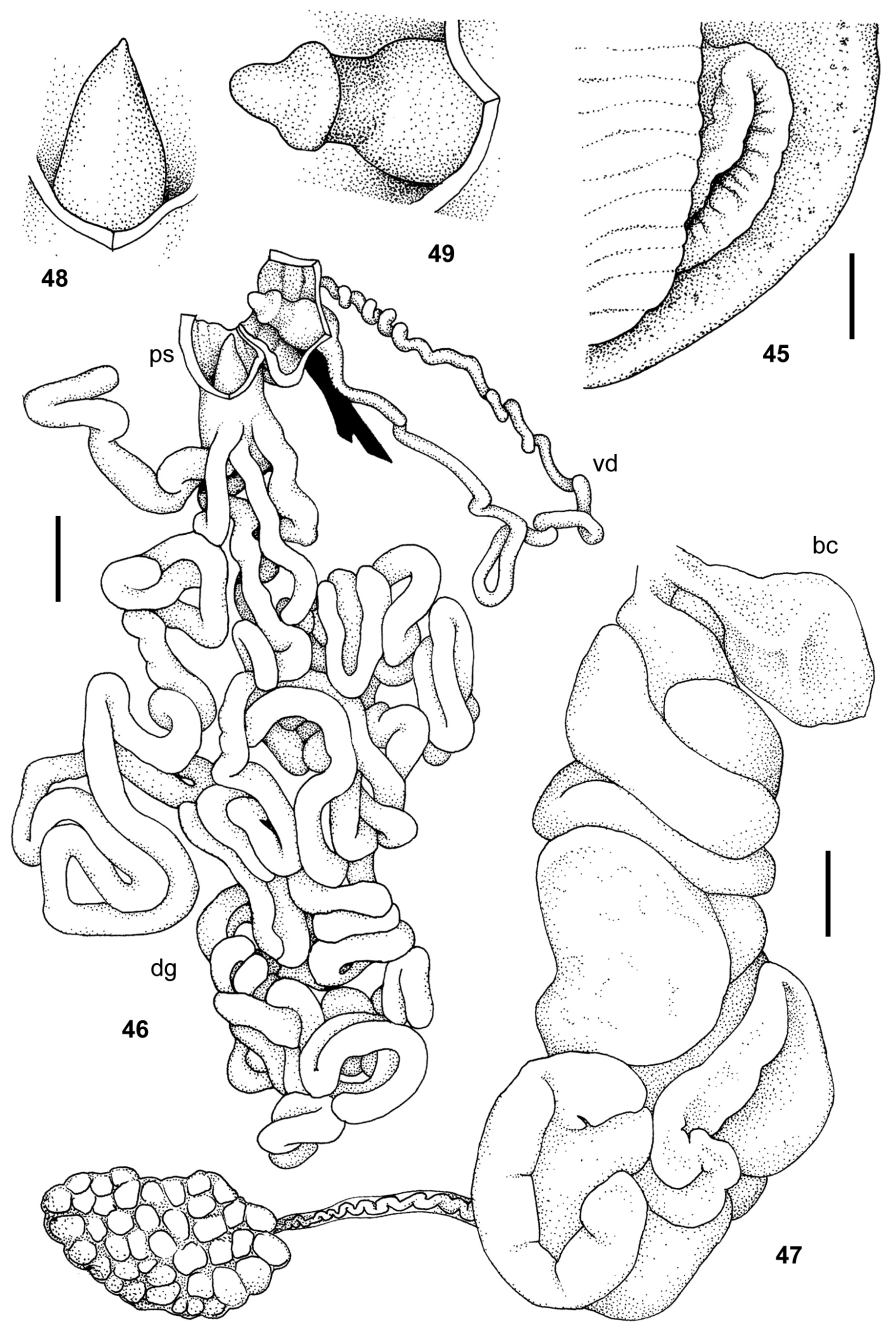
Pseudoveronicella (Hoffmannia) zootoca tanzaniensis subsp. n., holotype. **45** anus **46** male genitalia **47** female genitalia **48** tip of penial gland **49** penial verge. All scale bars 2 mm.

Jaw and radula (Figs 25, 39–41). Jaw with minor median projection, 1.9 mm wide. Radula with central tooth and up to 70 lateral and marginal teeth in a half-row, in over 100 rows. Lateral teeth tricuspid, with mesocones pointed and largest; many outermost marginals with serrated outer edges.

Genitalia (Figs 66–67). Visceral cavity almost reaches tail (only the posterior 10% of body solid). No stimulator. Penial complex consisting of: stout free penis, narrow in its straight proximal part, with one (holotype) and two (paratype) volutions; epiphallus 1 a little shorter than penis; epiphallus 2 extremely short; long epiphallic caecum; pyriform calc sac. Penial retractor muscle arising from diaphragm. Internally, penis tube thick walled. No penis verge. Penis sheath thick walled, free in its proximal half, fused with penial tube in its distal half. Atrium relatively wide, with internal longitudinal folds. Vagina short, with a thick muscular wall all around, with a narrow lumen. Duct of bursa copulatrix long; bursa elongated. Oviductal gland quite voluminous. Oviductus very short and broad. Vas deferens short. Ovotestis sited posterior to albumen gland.

Spermatophores (Figs 68–70). In paratype two empty spermatophores found in the bursa entangled with a third, even more corroded one. Length +/- 11 mm. Apex pointed. Outer side of ampulla with numerous, scattered very tiny pointed nodules; interior side and apical part smooth; very short tail with two rows of very tiny hooks, not exceeding 0.03 mm.

########## Etymology.

Named in honour of Mary Seddon, in recognition of her work, encouragement and support of others in the study of East African terrestrial molluscs, and in mollusc conservation worldwide.

########## Distribution and habitat.

Probably endemic to forest in the Uluguru Mts., where several other endemic forest molluscs occur (e.g. Verdcourt 2006; Tattersfield and Rowson 2011).

########## Remarks.

*Tanzalimax seddonae* sp. n. resembles *T. tattersfieldi* sp. n. in the striking appearance of the penial complex, the penis being very muscular in both the penial tube and the penial sheath, and in the very short epiphallus 2. The vagina is also strikingly muscular. The species differs from *T. tattersfieldi* sp. n. by the much smaller genital system, despite both species having a similar body length. The penis has only 1–2 volutions instead of 5 in the latter. The vagina has a muscular ring, while in the latter species it is a muscular bulb at one side. The spermatophore looks smooth at first glance, but has numerous scattered very tiny pointed nodules at the outer side of the ampulla; the very short tail has two rows of very tiny hooks. In preservation the body looks warty and is uniformly ivory coloured, lacking markings.

**Figures 50–54.**
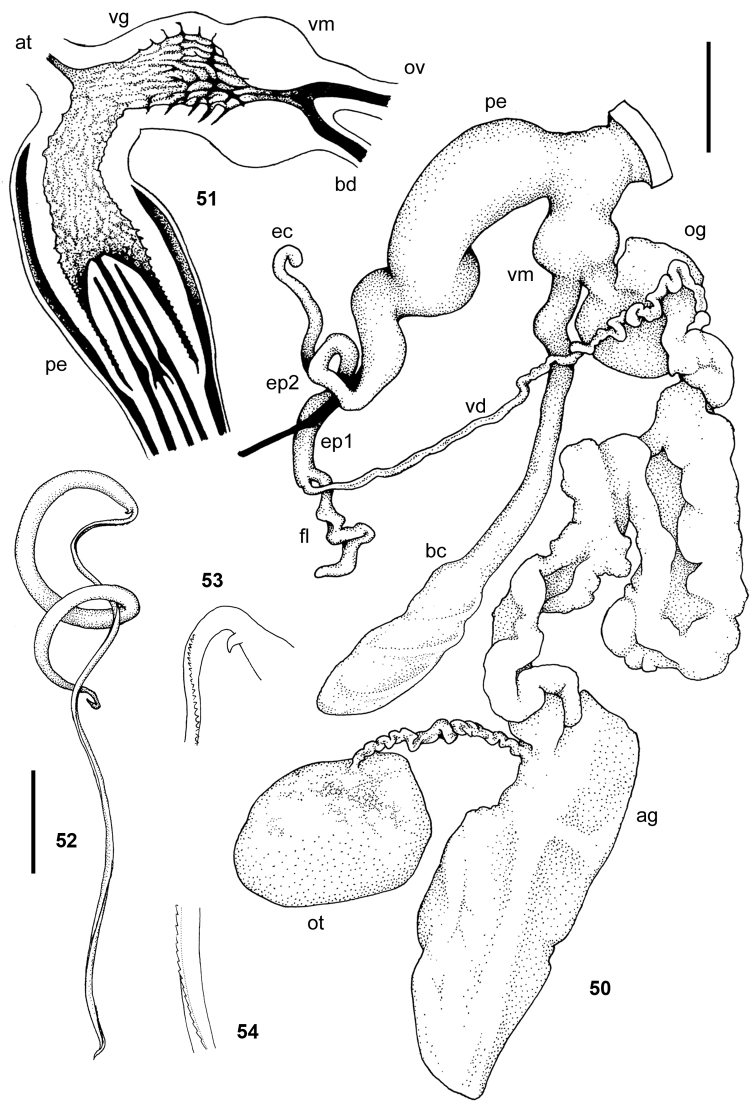
*Dendrolimax parensis* sp. n., holotype. **50** genitalia **51** interior of penis and vagina **52** spermatophore **53–54** details of angle and tail serration of spermatophore. All scale bars 5 mm.

######### Genus Udzungwalimax gen. n.

Taxon classificationAnimaliaStylommatophoraUrocyclidae

http://zoobank.org/6D8C31D2-35FE-4D1F-9997-0FEBD39EC153

########## Type species.

*Udzungwalimax suminis* sp. n.

########## Included species.

*Udzungwalimax suminis* sp. n.

**Figures 55–58.**
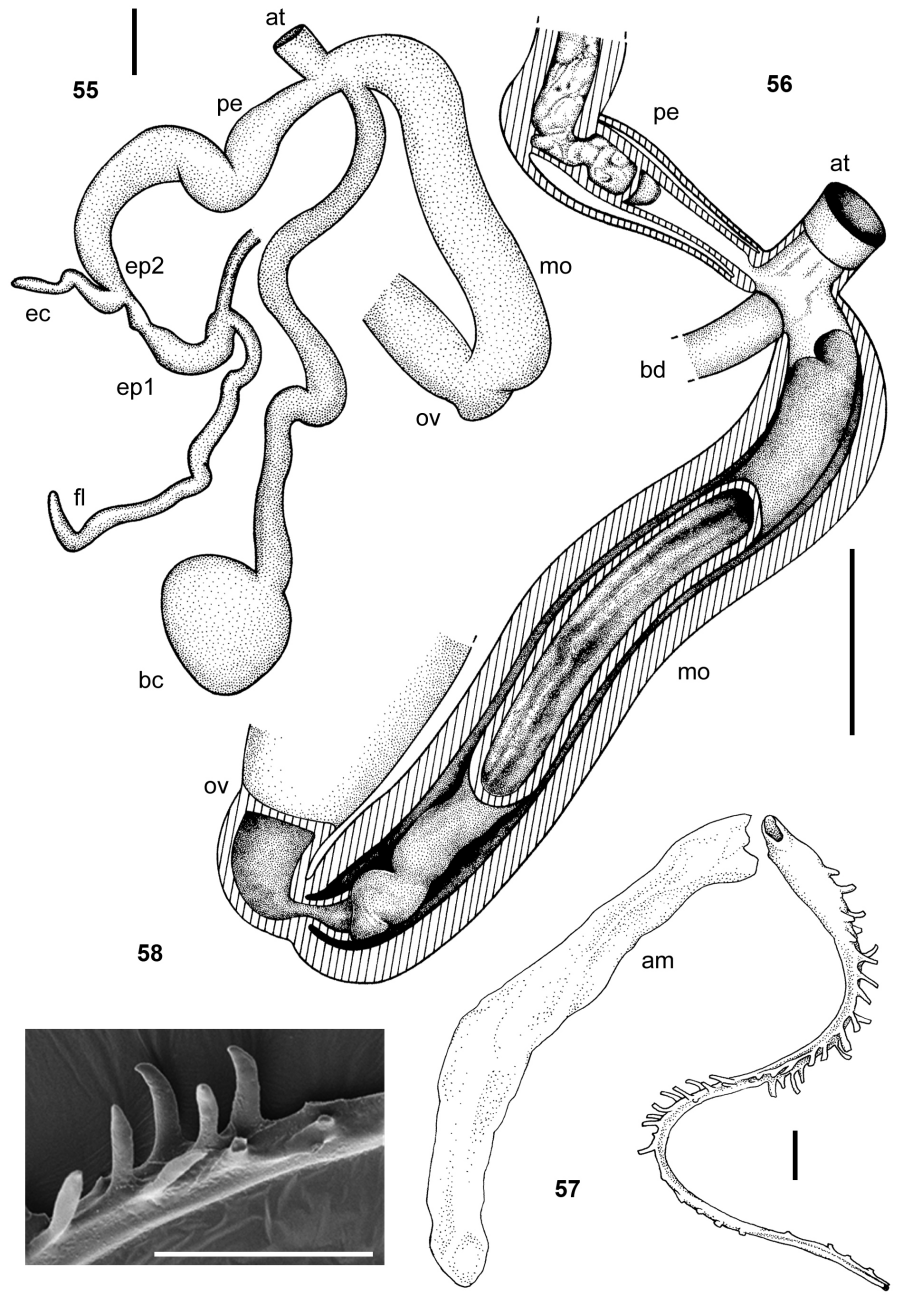
*Leptichnoides avisexcrementis* sp. n., Paratype 4. **55** genitalia **56** interior of penis and vagina **57–58** *Leptichnoides avisexcrementis* sp. n., holotype **57** spermatophore **58** details of tail serration of spermatophore. Scale bars 1 mm (**55–56**), 100 μm (**57–58**).

########## Diagnosis.

Slug belonging to tribe Urocyclini. Genital apparatus without an accessory organ (atrial diverticulum, sarcobelum or dart sac); penis strong, with straight proximal part, narrowing towards the twisted distal part; penial tube thick-walled with broad lumen; penial sheath very thin. Vagina thick-walled with a distinct muscular part at one side; oviductal gland present, entering the vagina at a sharp angle. Spermatophore smooth. Radula: median and lateral teeth tricuspid, outermost laterals and marginals bicuspid.

########## Etymology.

From the Udzungwa Mts. and Latin *limax*, a slug (no relationship to *Limax* Linnaeus, 1758 is implied).

########## Known distribution.

Forest in the Udzungwa Mts. of the Eastern Arc, Tanzania.

########## Gender.

Masculine.

########## Remarks.

See also remarks on *Atrichotoxon*, *Tanzalimax* gen. n., and *Phaneroporus* above. The schematics of the structure of the genitalia (Figs 74–75) can be contrasted with those in Van Goethem (1977: 28).

**Figures 59–63.**
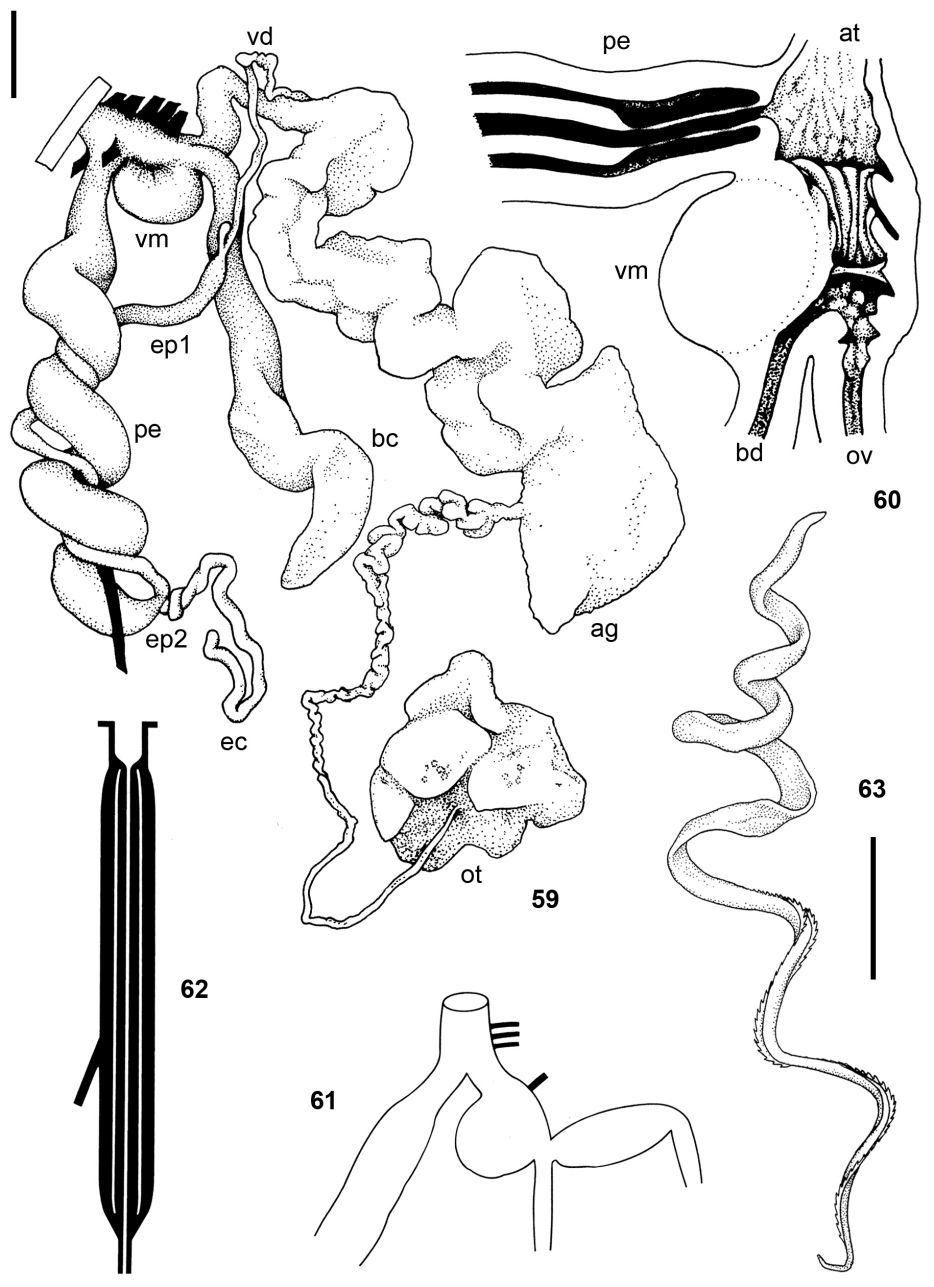
*Tanzalimax tattersfieldi* gen. & sp. n. **59–62** holotype **59** genitalia **60** interior of penis and vagina **61–62** schematics of genitalia and penis **63** Paratype 2, spermatophore. All scale bars 2 mm.

######### Udzungwalimax suminis sp. n.

Taxon classificationAnimaliaStylommatophoraUrocyclidae

http://zoobank.org/38EBC26E-69DB-44D0-A51E-2E266AE69B2F

Figs 18–20 , 26 , 42–44 , 71–75

########## Material.

TANZANIA: Holotype NMW.Z.1997.007.00011: 1 ad., Lulanda FR (8.62°S, 35.62°E), Mufindi District, montane forest at 1430 m alt., leg. AK, NO, CFN, MBS & PT, 12 Feb. 1997 (sample IG). Paratype 1 NMW.Z.1997.007.00013: 1 ad., data as previous but sample IH. Paratypes NMW.Z.1997.007.00014: 4 ads., 1 juv.?, data as previous. Paratype RBINS.I.G. 33548/MT.3611: 1 ad., data as previous. Paratype NMT: 1 ad., data as previous. Paratypes NMW.Z.1997.007.00012: 2 juvs., data as previous but sample IG.

########## Description.

External appearance (Figs 18–19). (In preservation; living appearance not recorded). Medium-sized (46 mm long) slug, body, head and tentacles rich brown, lacking markings save for a few small scattered dark brown spots on and around the mantle. Sole coloured as body, tripartite. Moderate dorsal keel along whole length of tail, terminating in a short, blunt caudal appendage. Supraperipodial groove not evident. Tail and flanks with moderately large, but smooth and very flat tubercules. Mantle moderately sized (approx. 35% of body length) with finely granular surface and large shell pore, attached at rear. Two probable juveniles, collected with the holotype, have additional dark brown markings on and around the mantle.

Shell (Fig. 20). Fingernail-shaped, nearly symmetrical, 7.3 mm long, thin and weakly mineralised, with periostracum just extending beyond the margins.

Jaw and radula (Figs 26, 42–44). Jaw with strong median projection. Radula with central tooth and up to 66 lateral and marginal teeth in a half-row, in over 100 rows. Lateral teeth tricuspid, outermost laterals becoming bicuspid, but with mesocones pointed and largest. No serrated outer edges to the outermost marginals.

Genitalia (Figs 71–74). Visceral cavity almost reaches tail (only the posterior 10% of body solid). No stimulator. Penial complex consisting of: stout free penis, broad in its proximal part then rapidly narrowing; epiphallus 1 a little shorter than penis, epiphallus 2 short; long epiphallic caecum; pyriform calc sac. Penial retractor muscle arising from diaphragm. Internally, penis covered with many rounded, mamillate papillae with hard tips, arranged in irregular rows, some on a tongue-like flap. Papillae replaced by horizontal folds or pilasters at point where free penis narrows. No obvious verge in penis, but a basal constriction present. Very thin, transparent penial sheath present. Atrium long, with weak internal folds. Vagina with a muscular swelling at one side, internally with strong irregular folds. Bursa copulatrix duct robust, very long, not pigmented or ornamented, internally with weak longitudinal pilasters; bursa voluminous, thin-walled, pointed apically. Oviductal gland small, oviductus short and broad, leaving vagina at a sharp angle. Vas deferens short. Ovotestis sited posterior to albumen gland.

Spermatophores (Fig. 75). Two spermatophores from bursa of holotype, up to 18 mm long when coiled, with 4.5 volutions. No clear division between ampulla and tail. Spermatophore pointed at both ends, appearing completely smooth, without obvious hooks or keels; cylindrical, although becoming slightly more laterally compressed towards tail tip.

**Figures 64–65.**
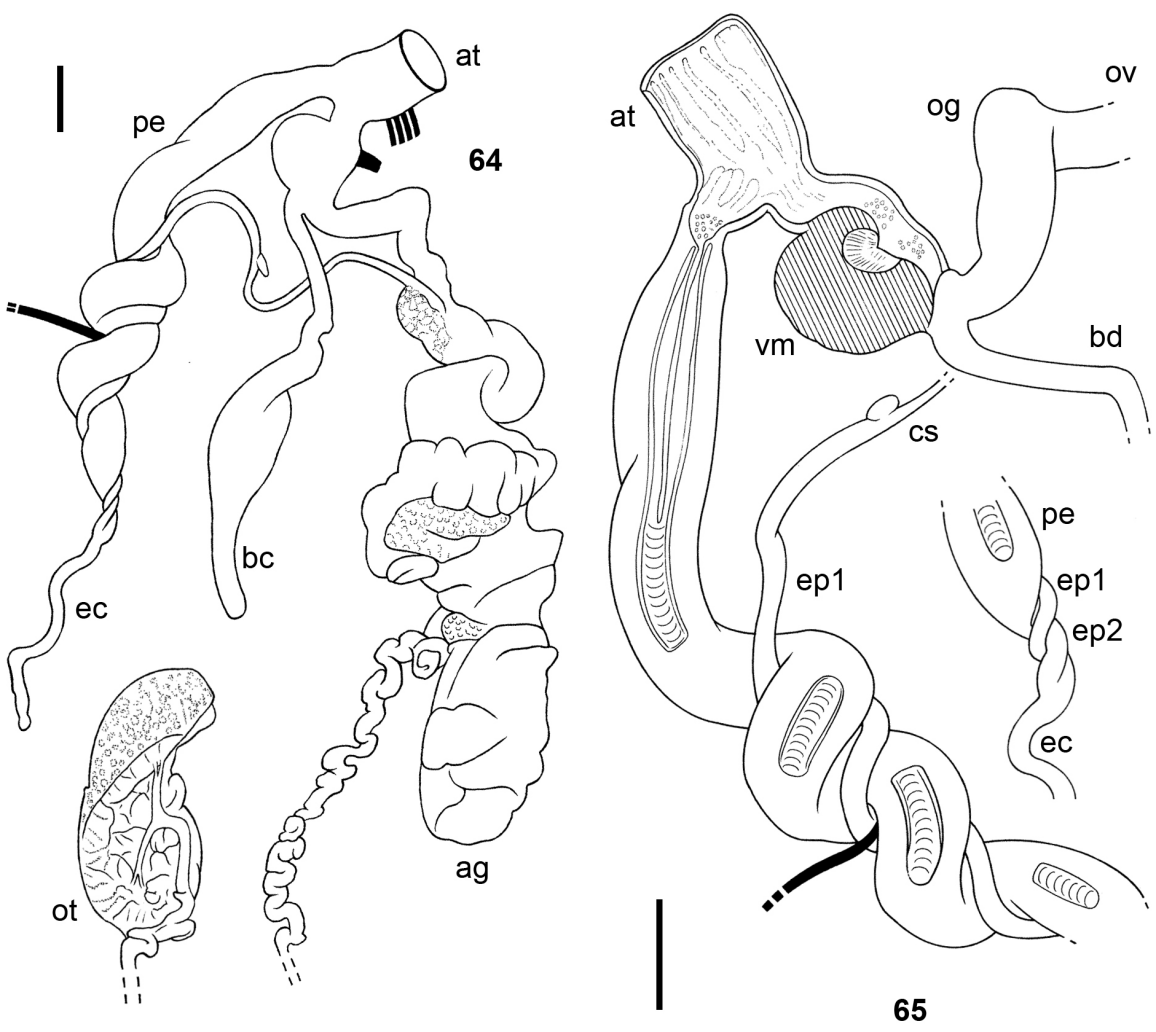
*Tanzalimax tattersfieldi* gen. & sp. n., Paratype 1. **64** genitalia **65** interior of penis and vagina. All scale bars 2 mm.

########## Etymology.

From Latin ‘suminis’, a sow’s udders, or a breeding sow, used as a noun in apposition referring to the mamillate surface of the penis.

########## Distribution and habitat.

Probably endemic to forest in the Udzungwa Mts., where several other endemic forest molluscs occur (Rowson & Van Goethem 2012).

########## Remarks.

This species is apparently unique in its hard-tipped, mamillate papillae inside the penis. No similar structures are mentioned or illustrated in Van Goethem (1977) or are present in any other species examined. They are not the same structures as the “lobules” inside the atrium of *A. usambarense* (Verdcourt & Polhill 1961, Van Goethem 1977). Neither are they present in *Emphysetes* Verdcourt, 2003, another recently described genus of Urocyclini from Udzungwa (Verdcourt 2003; observation verified from the holotype by A. J. de Winter, Leiden). The most similar structures seem to be the small, easily dislodged, irregular spines found on the penial prepuce of *Polytoxon robustum* (Simroth, 1896) (Van Goethem 1977), or perhaps the splinter-like crystals described by Verdcourt and Polhill (1961) in their *Atrichotoxon* “sp. n.” from the Taita Hills. Van Goethem (1973) described tubercles inside the penis of the the Malawian *Atoxonoides aberrans* Van Goethem, 1973, but they are not mamillate in shape and were not said to have hard tips.

###### Superfamily Limacoidea Lamarck, 1801

####### Family Agriolimacidae Wagner, 1935

######## Genus *Deroceras* Rafinesque, 1820

######### Deroceras cf. laeve

Taxon classificationAnimaliaStylommatophoraAgriolimacidae

(Müller, 1774)

########## Material.

TANZANIA: NMW.Z.2001.040.00001: 2 ads., central Mbeya (8.91°S, 33.46°E), Mbeya District, in a cabbage from town market, approx. 1600-1800 m alt., leg. MBS, PT & AR, 25 Jun. 2001. The cabbage, probably locally grown, also harboured a juvenile *Elisolimax* or *Bukobia* sp. (NMW.Z.2001.040.00002). Comparative material of non-African *Deroceras* spp.: specimens cited in Rowson et al. 2014a; b).

########## Remarks.

The genus *Deroceras* is primarily Palaearctic, but nonetheless is represented by a few species in Ethiopia. It includes several species spread widely by humans. These include the pest *D. reticulatum* (Müller, 1774) and the “tramp slug” *D. invadens* Reise, Hutchinson, Schunack & Schlitt, 2011 (see Reise et al. 2011 for synonymy). Although both species and *D. laeve* (Müller, 1774) are well-established in South Africa (Herbert 2010), records of *Deroceras* in tropical Africa are few. Verdcourt (1960a) recorded *D. laeve andecolum* (D’Orbigny, 1837) from a Nairobi garden. He later listed *D. laeve* from Muguga and Ruiru, both near Nairobi, and from Thika where it was damaging orchids, later listing it from the “Nairobi area” generally (Verdcourt 1965, 2006). Nairobi and Mbeya have relatively similar, cool climates when compared to “Zanzibar”, from which two *Deroceras* have been reported: *D. laeve* (Simroth 1895) and *D. reticulatum* (Ellis 1969). The *D. laeve* record seems plausible, given that species’ apparently very broad ecological tolerance (Wiktor 2000), although there is some evidence *D. laeve* may comprise more than one species (Rowson et al. 2014a). Rowson (2007) considered the *D. reticulatum* record doubtful and to require confirmation. *Deroceras invadens* has now been intercepted on Kenyan flowers arriving in the USA (Hutchinson et al. 2014). These authors reported *D. laeve* from São Tomé, where it had been identified as *D. invadens*, but also suggest that some records of “*D. laeve*” outside Europe may refer to *D. invadens*. *Deroceras laeve* was also reported from Ethiopia by Simroth (1895) along with *Agriolimax jickelii* “Heynemann”, a species Wiktor (2000) considers a nomen dubium. Simroth later (1904) described 14 other *Deroceras* species from Ethiopia. Some could potentially be confused with introduced species, but others are highly distinctive and doubtless endemic. Wiktor (2000) maintained ten of them in his revision. According to Wiktor (2000) the most southerly native occurrence of Agriolimacidae is *D. uataderensis* (Simroth, 1904), described from Lake Gandjule in the southern Ethiopian Rift (Lake Abaya or Lake Chamo, approx. 6°N, approx. 1200 m alt.). Terrestrial molluscs with apparently Ethiopian or Palaeartic links are known from the archipelago-like Afroalpine and Afromontane regions isolated on the highest East African mountains (e.g. Tattersfield et al. 2001, Verdcourt 2006). However, on a broader scale, and at more moderate altitudes, the Ethiopian biota is biogeographically very distinct from that of southern Tanzania (e.g. Linder et al. 2012).

**Figures 66–70.**
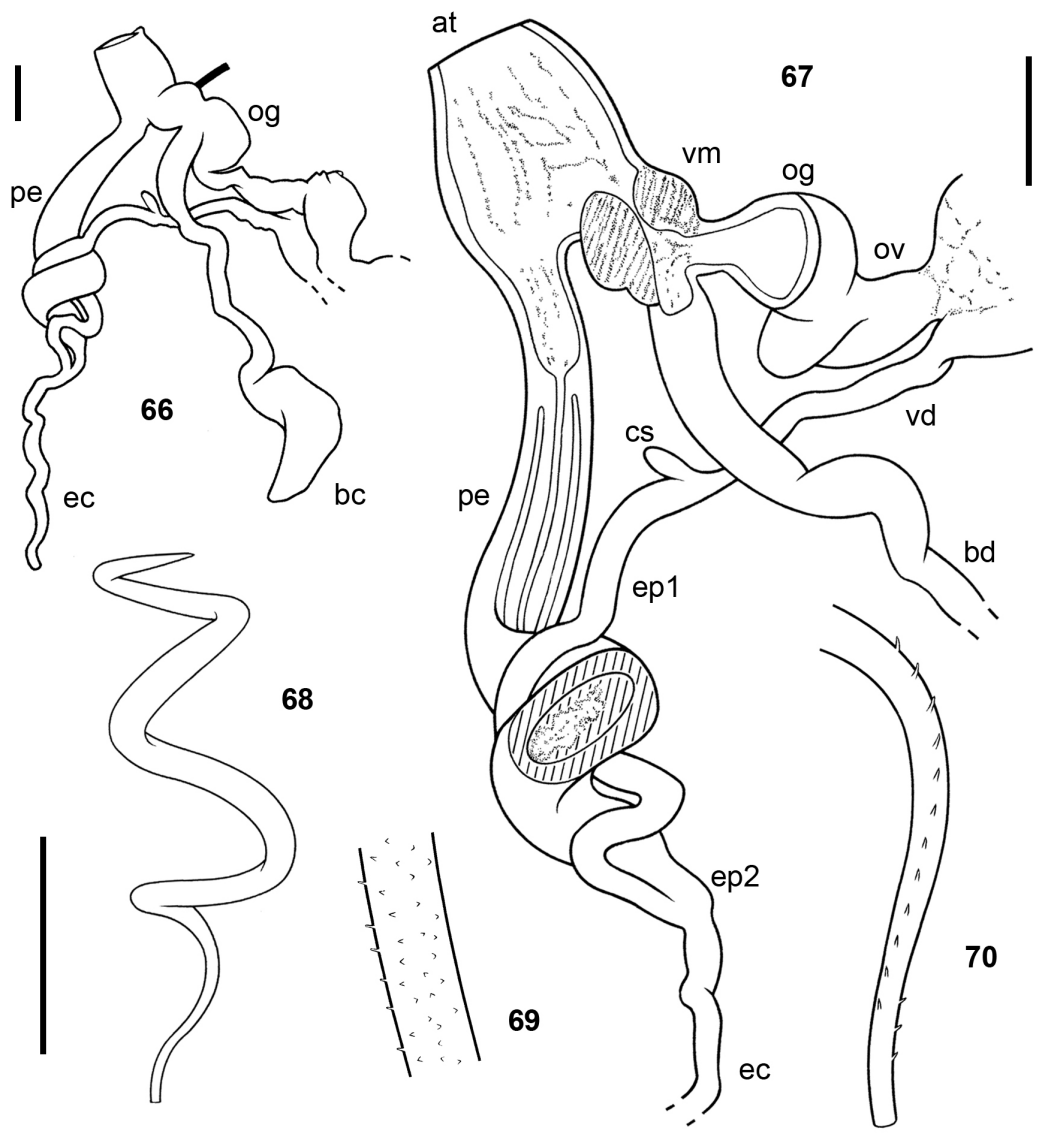
*Tanzalimax seddonae* gen. & sp. n., holotype. **66** genitalia **67** interior of penis and vagina **68** spermatophore **69–70** details of head and tail serration on spermatophore. All scale bars 2 mm.

The Mbeya slugs are 17.5 and 16.8 mm long, larger than most of the preserved *D. laeve* examined but smaller than most *D. invadens*, so in fact within the range of overlap. They resemble *D. reticulatum* (and some Ethiopian species) in being pale cream with black-brown tentacle retractors, and a dusting of light brown pigment along the centre of the mantle and forming a network between the tubercles at the top of the tail. The skin is thin, with the part of the mantle underlain by the shell relatively obvious. The pneumostome is surrounded by a contrastingly pale ring. The tail tip is steeply truncate. The skin, pneumostome and tail features are often considered diagnostic of *D. invadens* or *D. laeve* as compared to *D. reticulatum*, as is the length of the tail, although none may be infallible (e.g. Rowson et al. 2014b and references therein). Indeed, Herbert (2010) notes that some *D. invadens* in South Africa closely resemble *D. reticulatum* externally. Internally, no rectal caecum was found in the Mbeya slugs, ruling out *D. reticulatum* which has a large one (Wiktor 2000). This also would seem to rule out a group of taxa including *D. invadens*, which Wiktor treats as having a shallow, pocket-like caecum. However, it is clear that the caecum can be so shallow as to be undetectable in *D. invadens* (Quick 1960, Reise et al. 2011). The ovotestis lies relatively far forward, anterior of the rectum, and is scarcely exposed. Quick (1960) showed an anterior ovotestis for *D. invadens* and described a “less exposed” ovotestis for *D. laeve*. The female genitalia are well developed but the penis is reduced to a tiny nub without a retractor muscle (an aphallic condition). The combination of aphally and no rectal caecum makes the Mbeya slugs key to *D. laeve* in Wiktor (2000); indeed, aphally has often been used to attribute putative *D. invadens* specimens to *D. laeve* (e.g. Quick 1960, Wiktor 2000, Reise et al. 2006, Hutchinson et al. 2014). Although it has been suggested that *D. invadens* could potentially be aphallic (de Winter 1988), there is as yet no substantiated report of aphally in any *Deroceras* species other than *D. laeve* (J.M.C. Hutchinson pers. comm. 2017). Genetic data also suggest that worldwide *D. laeve* might consist of more than one species (e.g. Rowson et al. 2014a). We therefore attribute the Mbeya slugs to D. cf. laeve, a matter that could be settled with molecular data from this population. Until then any evidence of the spreading of non-native slugs in tropical Africa seems worth reporting, given the potential economic and conservation implications.

**Figures 71–75.**
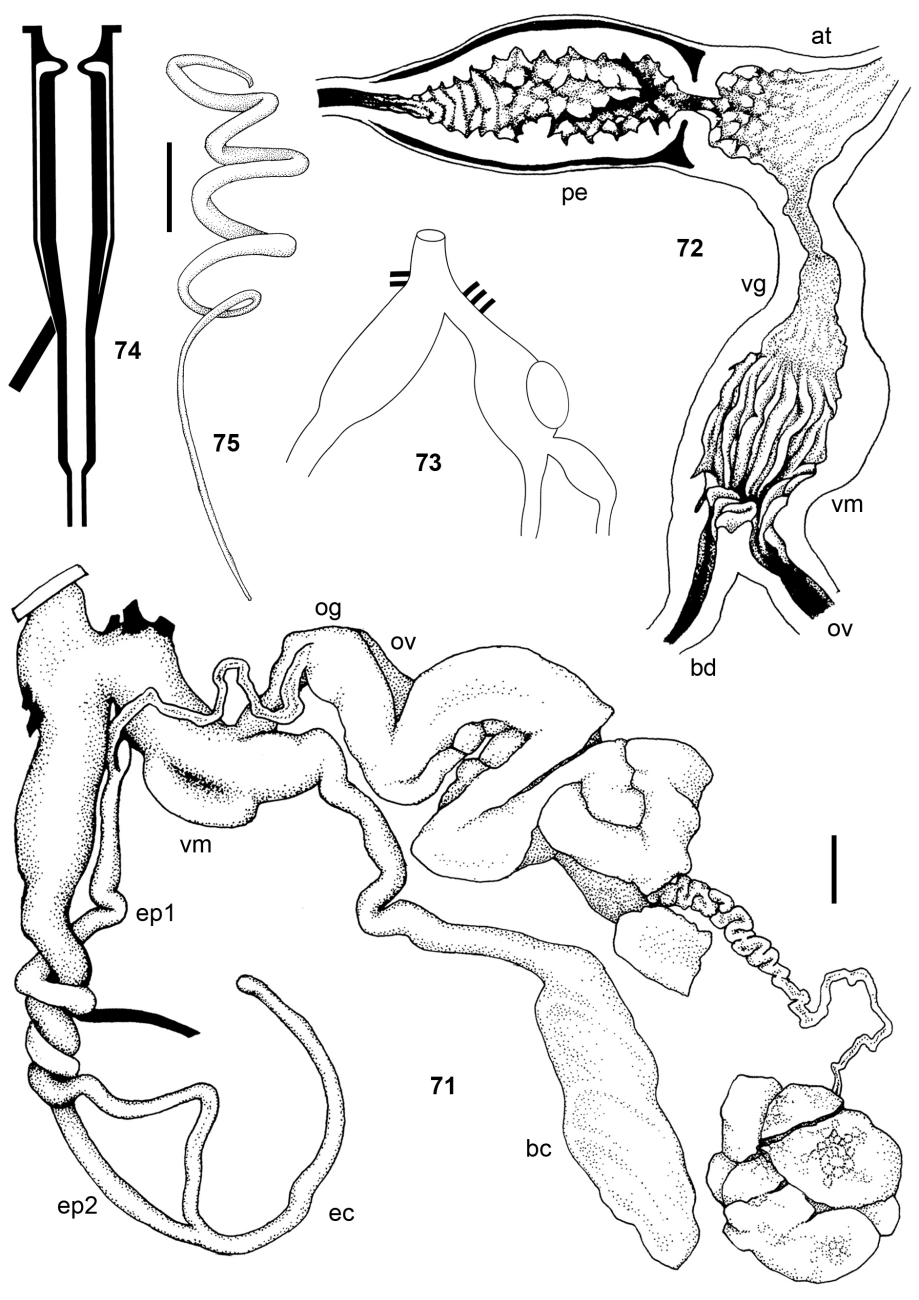
*Udzungwalimax suminis* gen. & sp. n., Paratype 1. **71** genitalia **72** interior of penis and vagina **73–74** schematics of penis and genitalia **75** spermatophore. All scale bars 2 mm

## Supplementary Material

XML Treatment for Pseudoveronicella (Hoffmannia) zootocatanzaniensis

XML Treatment for Dendrolimax leprosus

XML Treatment for Dendrolimax parensis

XML Treatment for Leptichnoides avisexcrementis

XML Treatment for Tanzalimax

XML Treatment for Tanzalimax tattersfieldi

XML Treatment for Tanzalimax seddonae

XML Treatment for Udzungwalimax

XML Treatment for Udzungwalimax suminis

XML Treatment for Deroceras cf. laeve

## References

[B1] Bouchet P Rocroi J-P (2005) Classification and nomenclator of gastropod families. Malacologia 47(1/2): 1–397.

[B2] Ellis AE (1969) British snails: the non-marine Gastropoda of Great Britain and Ireland. Clarendon Press, Oxford, 325 pp.

[B3] Forcart L (1953) The Veronicellidae of Africa (Mollusca, Pulmonata). Annales du Musée Royal du Congo Belge, Sciences Zoologiques 23: 1–110.

[B4] Forcart L (1967) Studies on the Veronicellidae, and Urocyclidae (Mollusca) of South Africa. Annals of the Natal Museum 18(3): 505–570.

[B5] Gerlach J (2006) Terrestrial and freshwater Mollusca of the Seychelles islands. Backhuys, Leiden, The Netherlands, 141 pp.

[B6] Glaubrecht M (2010) Slug(-gish) scheince, or an annotated catalogue of the types of tropical vaginulid and agriolimacid pulmonates (Mollusca, Gastropoda), described by Heinrich Simroth (1851–1917), in the Natural History Museum Berlin. Zoosystematics and Evolution 86(2): 315–335. https://doi.org/10.1002/zoos.201000014

[B7] Glaubrecht M Zorn C (2012) More slug(gish) science: another annotated catalogue on types of tropical pulmonate slugs (Mollusca, Gastropoda) in the collection of the Natural History Museum Berlin. Zoosystematics and Evolution 88(1): 33–51. https://doi.org/10.1002/zoos.201200005

[B8] Herbert DG (1997) The terrestrial slugs of KwaZulu-Natal: diversity, biogeography and conservation (Mollusca: Pulmonata). Annals of the Natal Museum 38: 197–239.

[B9] Herbert DG (2010) The introduced terrestrial Mollusca of South Africa. SANBI Biodiversity Series 15. South African National Biodiversity Institute, Pretoria, South Africa, 108 pp.

[B10] Herbert DG Mitchell A (2009) Phylogenetic relationships of the enigmatic land snail genus *Prestonella*: the missing African element in the Gondwanan superfamily Orthalicoidea (Mollusca: Stylommatophora). Biological Journal of the Linnean Society 96: 203–221. https://doi.org/10.1111/j.1095-8312.2008.01109.x

[B11] Hutchinson JMC Reise H Robinson DG (2014) A biography of an invasive terrestrial slug: the spread, distribution and habitat of *Deroceras invadens*. Neobiota 23: 17–64. https://doi.org/10.3897/neobiota.23.7745

[B12] Linder HP de Klerk HM Born J Burgess ND Fjeldså J Rahbek C (2012) The partitioning of Africa: statistically defined biogeographical regions in sub-Saharan Africa. Journal of Biogeography 39: 1189–1205. https://doi.org/10.1111/j.1365-2699.2012.02728.x

[B13] Muratov IV (2010) Terrestrial molluscs of Cabo Delgado and adjacent inland areas of north-eastern Mozambique. African Invertebrates 51(2): 255–288. https://doi.org/10.5733/afin.051.0203

[B14] Pollonera C (1906) Spedizione al Ruwenzori di S. A. R. Luigi Amedo di Savoia duca degli Abruzzi. VII. Vaginulidae e Urocyclidae. Bolletino dei Musei di Zoologia ed Anatomia comparata della R Universita di Torino 21(543): 1–6.

[B15] Pollonera C (1909) Molluschi: Stylommatophora. Spedizione al Ruwenzori di S. A. R. il Principe L. Amedeo di Savoia. Parte Scientifica 1: 181–205.

[B16] Pollonera C (1911) New species of Urocyclidae from British East Africa. Annals and Magazine of Natural History (Series 8) 8: 331–334.

[B17] Quick HE (1960) British Slugs (Pulmonata; Testacellidae, Arionidae, Limacidae) Bulletin of the British Museum (Natural History) Zoology 6: 103–226.

[B18] Reise H Hutchinson JMC Robinson DG (2006) Two introduced pest slugs: *Tandonia budapestensis* new to the Americas, and *Deroceras panormitanum* new to the Eastern USA. Veliger 48: 110–115.

[B19] Reise H Hutchinson JMC Schunack S Schlitt B (2011) *Deroceras panormitanum* and congeners from Malta and Sicily, with a redescription of the widespread pest slug as *Deroceras invadens* n. sp. Folia Malacologica 19: 201–233. https://doi.org/10.2478/v10125-011-0028-1

[B20] Rowson B (2007) Land molluscs of Zanzibar island (Unguja), Tanzania with the description of a new species of *Gulella* (Pulmonata: Streptaxidae). Journal of Conchology 39(4): 425–466.

[B21] Rowson B Tattersfield P (2013) Revision of *Dadagulella* gen. n., the “*Gulella radius*” group (Gastropoda: Streptaxidae) of the eastern Afrotropics, including six new species and three new subspecies. European Journal of Taxonomy 37: 1–46. https://doi.org/10.5852/ejt.2013.37

[B22] Rowson B Van Goethem JL (2012) A second remarkable slug and a thin-shelled *Trochonanina* snail from the Udzungwa Mts., Tanzania (Stylommatophora: Helicarionoidea: Urocyclidae). Journal of Conchology 41: 239–247.

[B23] Rowson B Warren BH Ngereza CF (2010) Terrestrial molluscs of Pemba Island, Zanzibar, Tanzania, and its status as an “oceanic” island. ZooKeys 70: 1–39. https://doi.org/10.3897/zookeys.70.762 10.3897/zookeys.70.762 PMC3088446 21594041

[B24] Rowson B Anderson R Turner JA Symondson WOC (2014a) The slugs of Britain and Ireland: undetected and undescribed species increase a well-studied, economically important fauna by more than 20%. PLOS One 9(3): e91907. https://doi.org/10.1371/journal.pone.0091907 10.1371/journal.pone.0091907 PMC3989179 24740519

[B25] Rowson B Turner JA Anderson R Symondson WOC (2014b) Slugs of Britain and Ireland: identification, understanding and control. Field Studies Council, Shropshire, 136 pp.

[B26] Simroth H (1895) Nacktschnecken. In: Deutsche-Ost-Afrika IV. Die Thierwelt Ost-Afrikas, Wirbellose Thiere. Geographische Verlagshandlung Dietrich Reimer, Berlin, 1–23. [pls. 1–2]

[B27] Simroth H (1904) Ueber die von Herrn Dr. Neumann in Abessinien gesammelten aulacopoden Nackschnecken. Zoologische Jahrbucher (Abteilung fur Systematik, Geographie und Biologie) 19: 673–726. [pls. 39–42]

[B28] Simroth H (1905) Über zwei Mißbildungen an Nacktschnecken. Zeitschrift für wissenschaftliche Zoologie 82: 494–522. [pl. 29, figs 1–19]

[B29] Simroth H (1910) Lissopode Nacktschnecken von Madagaskar, den Comoren und Mauritius. Unter Berücksichtung verwandter Arten. In: Voeltzkow A (Ed.) Reise in Ostafrika in den Jahren 1903-1905, 2(5): 577–622. [textfigs 1–16, pls. 25–26]

[B30] Tattersfield P Paul CRC Allen JA (2001) *Columella* in sub-Saharan Africa: a range extension of over 4000 kilometres? Journal of Conchology 37: 281–284.

[B31] Tattersfield P Rowson B (2011) *Tanzartemon* gen. n., a new land snail genus (Gastropoda, Pulmonata, Streptaxidae) from Morogoro District, Tanzania. Basteria 75(1/3): 39–50.

[B32] Van Goethem JL (1973) *Atoxonoides aberrans*, gen. n., sp. n., du Malawi (Mollusca Pulmonata, Urocyclidae). Bulletin de l’Institut Royal des Sciences Naturelles de Belgique Biologie 49(8): 1–11.

[B33] Van Goethem JL (1977) Révision Systématique des Urocyclinae (Mollusca, Pulmonata, Urocyclidae). Annales Musée Royal de l’Afrique Centrale, Tervuren, Belgique. Series in-8o, Zoologie 218: 1–355. [figs 1–719, pls. I–IV]

[B34] Van Mol JJ (1970) Révision des Urocyclidae (Mollusca, Gastropoda, Pulmonata): anatomie, systématique, zoogéographie. Première partie. Annales Musée Royal de l’Afrique Centrale, Tervuren, Belgique Series in-8o, Zoologie 180: 1–234.

[B35] Verdcourt B (1960a) East African slugs of the family Urocyclidae. Journal of East African Natural History 23(102): 200–209. [fgs 1–4]

[B36] Verdcourt B (1960b) East African slugs of the family Urocyclidae (Part II). Journal of East African Natural History 23(103): 233–240. [figs 5–8]

[B37] Verdcourt B (1962) Report on a collection of East African Slugs (Urocyclidae). Journal of the East African Natural History Society 24(105): 29–41. [figs 1–14]

[B38] Verdcourt B (1965) Report on a further collection of East African slugs (Urocyclidae). Revue de Zoologie et de Botanique Africaines 71: 274–296. [figs 1–15]

[B39] Verdcourt B (1988) Collectors in East Africa - 13. F. Stuhlmann (part 1). Conchologists’ Newsletter 106: 113–117.

[B40] Verdcourt B (1989a) Collectors in East Africa – 13. F. Stuhlmann (part 2). Conchologists’ Newsletter 109: 181–187.

[B41] Verdcourt B (1989b) Collectors in East Africa – 13. F. Stuhlmann (part 3). Conchologists’ Newsletter 110: 211–219.

[B42] Verdcourt B (2003) *Emphysetes udzungwensis*, a most remarkable new genus and species of slug from the Udzungwa Mts. in southern Tanzania (Gastropoda, Pulmonata, Urocyclidae) Basteria 67(1-3): 107–111.

[B43] Verdcourt B (2004) New and little known species of terrestrial Mollusca from East Africa and Congo (Kinshasa). Annales Historico-Naturales Musei Nationalis Hungarici 96: 299–315. [figs 1–20]

[B44] Verdcourt B (2006) A revised list of the non-marine Mollusca of East Africa (Kenya, Uganda and Tanzania, excluding Lake Malawi). B. Verdcourt, Maidenhead, 75 pp.

[B45] Verdcourt B Polhill RM (1961) East African slugs of the family Urocyclidae, III & IV. The genus Trichotoxon. Journal of East African Natural History special supplement 7: 1–36. [figs 8b–42]

[B46] Wiktor A (2000) Agriolimacidae (Gastropoda: Pulmonata) – a systematic monograph. Annales Zoologici 49: 347–590.

[B47] de Winter AJ (1988) Remarks on the non-marine molluscan fauna of the Azores. Basteria 52: 105–109.

[B48] Wronski T Hausdorf B (2010) Diversity and body-size patterns of land snails in rain forests in Uganda. Journal of Molluscan Studies 76: 87–100. https://doi.org/10.1093/mollus/eyp048

